# Multiple recombination events between two cytochrome P450 loci contribute to global pyrethroid resistance in *Helicoverpa armigera*

**DOI:** 10.1371/journal.pone.0197760

**Published:** 2018-11-01

**Authors:** Thomas K. Walsh, Nicole Joussen, Kai Tian, Angela McGaughran, Craig J. Anderson, Xinghui Qiu, Seung-Joon Ahn, Lisa Bird, Nena Pavlidi, John Vontas, Jaeeun Ryu, Akhtar Rasool, Isabella Barony Macedo, Wee Tek Tay, Yongjun Zhang, Mary E. A. Whitehouse, Pierre Jean Silvie, Sharon Downes, Lori Nemec, David G. Heckel

**Affiliations:** 1 Black Mountain Laboratories, Commonwealth Scientific and Industrial Research Organisation, Acton, Australian Capital Territory, Australia; 2 Department of Entomology, Max Planck Institute for Chemical Ecology, Jena, Germany; 3 State Key Laboratory of Integrated Management of Pest Insects and Rodents, Institute of Zoology, Chinese Academy of Sciences, Beijing, China; 4 School of BioSciences, University of Melbourne, Melbourne, Victoria, Australia; 5 Biological and Environmental Sciences, University of Stirling, Stirling, United Kingdom; 6 Horticultural and Herbal Crop Environment Division, National Institute of Horticultural and Herbal Science, Suwon, Korea; 7 Tamworth Agricultural Institute, New South Wales Department of Primary Industry, Calala, New South Wales, Australia; 8 Department of Biology, University of Crete, Rethymno, Greece; 9 Laboratory of Pesticide Science, Agricultural University of Athens, Athens, Greece; 10 Insect Molecular Biology Laboratory, National Institute for Biotechnology and Genetic Engineering, Faisalabad, Pakistan; 11 Faculdade de Farmácia, Universidade Federal de Minas Gerais, Belo Horizonte, Minas Gerais, Brazil; 12 Institute of Plant Protection, Chinese Academy of Agricultural Sciences, Beijing, China; 13 Australian Cotton Research Institute, Narrabri, New South Wales, Australia; 14 Agroécologie et intensification durable des cultures annuelles, Centre de coopération internationale en recherche agronomique pour le développement, Montpellier, France; Plant and Food Research, NEW ZEALAND

## Abstract

The cotton bollworm, *Helicoverpa armigera* (Hübner) is one of the most serious insect pest species to evolve resistance against many insecticides from different chemical classes. This species has evolved resistance to the pyrethroid insecticides across its native range and is becoming a truly global pest after establishing in South America and having been recently recorded in North America. A chimeric cytochrome P450 gene, *CYP337B3*, has been identified as a resistance mechanism for resistance to fenvalerate and cypermethrin. Here we show that this resistance mechanism is common around the world with at least eight different alleles. It is present in South America and has probably introgressed into its closely related native sibling species, *Helicoverpa zea*. The different alleles of *CYP337B3* are likely to have arisen independently in different geographic locations from selection on existing diversity. The alleles found in Brazil are those most commonly found in Asia, suggesting a potential origin for the incursion of *H*. *armigera* into the Americas.

## Introduction

In agriculture, insecticides are commonly used to control insect pest species. However, pest management is hindered by the increasing level of insecticide resistance in various pest species worldwide. One of the most serious insect pest species to evolve resistance against many insecticides from different chemical classes is the Old World cotton bollworm, *Helicoverpa armigera* (Hübner). Its pest status is further strengthened by its highly polyphagous nature and extremely wide geographical distribution, with the latter due to its ability to migrate long distances [1–4]. Recent work confirmed a major incursion of *H*. *armigera* into Brazil, as well as Argentina, Uruguay and Paraguay and it has been detected in Puerto Rico and Florida [5–8].

Pyrethroid resistance of *H*. *armigera* was first reported in Australian populations in 1983, six years after the introduction of these insecticides [9]. Since then, pyrethroid resistance has been reported from various populations globally but, in most cases, the molecular resistance mechanism in *H*. *armigera* is yet to be determined. The two major pyrethroid resistance mechanisms common to other species are target site insensitivity, and metabolic resistance by cytochrome P450 monooxygenases (P450s) and carboxylesterases [10, 11]. Target site resistance against pyrethroids, also known as knockdown resistance (*kdr*), is based on one or more point mutations in the insect sodium channel protein, which is the target of pyrethroid insecticides. In *H*. *armigera*, two mutations (D1549V and E1553G) in the sodium channel protein have been described [12, 13]. In the case of metabolic resistance, 18 P450s have been identified as capable of metabolizing one or more pyrethroid insecticides after heterologous expression [11, 14–19]. One of them is the chimeric *CYP337B3*, recently identified in Australian *H*. *armigera* and thought to have arisen through an unequal crossing-over between the parental genes, *CYP337B1* and *CYP337B2* [18].

Joussen et al. [18] demonstrated that, after heterologous expression, *CYP337B3* is capable of metabolizing fenvalerate, a type II pyrethroid ester, to the nontoxic 4'-hydroxyfenvalerate. In contrast, the parental enzymes exhibit no detectable fenvalerate metabolism. *CYP337B3* has been shown to confer 42-fold resistance towards fenvalerate in Australian *H*. *armigera* lines differing solely in the presence of *CYP337B3* and its parental genes [18]. Similarly, a 49-fold resistance factor was reported by Forrester et al. [20] for an Australian field-collected population, indicating that metabolism of fenvalerate by *CYP337B3* is the detoxification mechanism *in vivo*.

Recently, *CYP337B3* was also identified in field-collected populations from Pakistan [21] and China [22], confirming its presence outside Australia. Sequence analysis revealed a distinct *CYP337B3* allele (*CYP337B3v2*), in the Pakistani population and three distinct alleles in the Chinese populations (*CYP337B3v2*, *CYP337B3v3*, *CYP337B3v4*) that differ from the Australian allele (*CYP337B3v1*), by a number of synonymous and non-synonymous SNPs, in addition to variability of the intron sequence and size. This variation may result from different crossing-over positions during recombination of the *CYP337B1* and *CYP337B2* parental genes, with different alleles of *CYP337B1* and *CYP337B2* involved in the crossing-over. Such a pattern is indicative of independent origins of the various *CYP337B3* alleles, however further work would be required to determine whether this is accurate across a wide geographic distribution of samples. Given the high frequency of this allele around the world, it is possible that the population of *H*. *armigera* now established in South America [5, 23, 24] may be carrying this allele and that the possession of *CYP337B3* may confer a selective advantage over local species.

Previous studies with allozymes and mitochondrial DNA indicated that very little population structure among *H*. *armigera* populations, though more recent work using many thousand genomic markers has shown that the population of *H*. *armigera* in Australia can be differentiated from the rest of the world [25, 26]. This lack of structure makes the identification of the *CYP337B3* chimeric origin difficult, as well as more generally hampering the identification of source populations in the case of incursions into the New World. However, examining patterns of distribution and diversity among recently selected genes can shed light on such processes. *CYP337B3* is a strong candidate for having undergone rapid selection due to its role in pyrethroid resistance, which has a short (approximately 50 year) history across much of the globe [27].

Here, we use field-collected populations from 17 countries distributed through Africa, Asia, Oceania, Europe, and South America to first examine the evolutionary history of the chimeric *CYP337B3* gene and the global spread in *H*. *armigera*. We use global allelic frequency data to show that *CYP337B3* is commonly found around the world, with at least six likely independent unequal crossing-over events giving rise to the same resistance phenotype in different regions. We also confirm recent data showing evidence of hybridisation between invasive *H*. *armigera* from Brazil and the local *Helicoverpa zea* population, including the exchange of the *CYP337B3* gene [26, 28]. Furthermore, we then use the different *CYP337B3* alleles to shed light on the nature of selection of *CYP337B3*, finding evidence to support recent selection operating on standing variation as opposed to novel mutations arising as a result of pyrethroid application.

## Materials and methods

### Population samples

**Fig 1:**
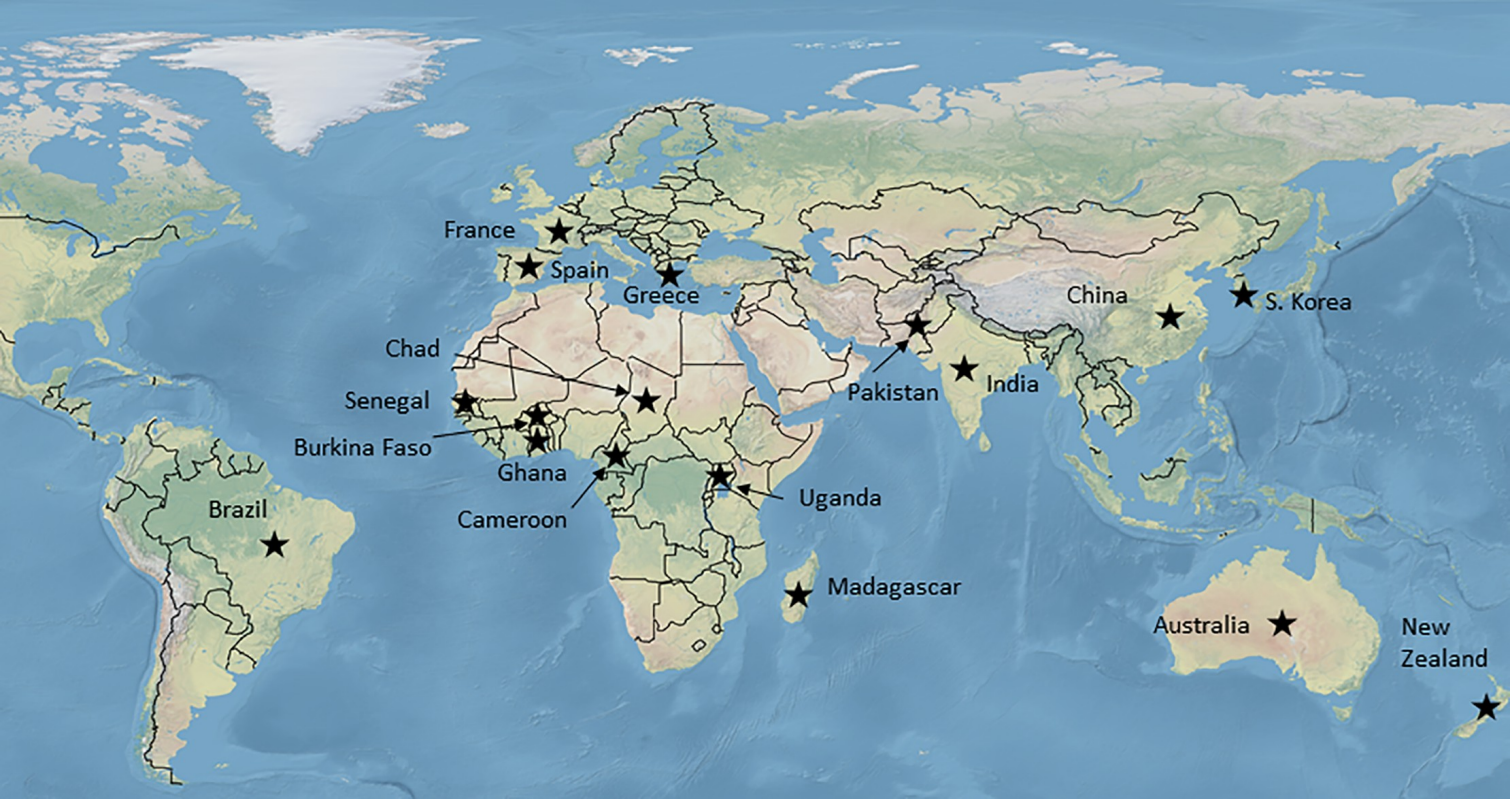
Collection localities. *H*. *armigera* (n = 986) were sampled from 17 countries (black stars): Australia, New Zealand, China, Korea, India, Pakistan, Greece, Spain, France, Burkina Faso, Cameroon, Chad, Ghana, Madagascar, Senegal, Uganda, and Brazil. *H*. *zea* were sampled from Brazil (n = 43) and the United States of America (n = 16). Modified from public domain map from Natural Earth (http://www.naturalearthdata.com).

A total of 1063 insect samples were used in the current study, including several specimens collected and used in previous work [5, 25, 26, 28, 29]. *H*. *armigera* was collected from 17 countries, *H*. *zea* from two (Fig 1; S1 Table). All insect samples were collected from agricultural areas with the permission of the landholder or by trapping using species-specific pheromones. No collecting permits were required because both species are common agricultural pests. No endangered or protected species were used in this work. Samples were usually collected as larvae from wild and crop host plants, as adult moths via light/pheromone traps or as larvae after bioassay. For one set of samples from China (n = 240), larvae were collected from colonies derived from eight recently collected field samples (established with 20 to 50 individuals). Collection dates range from 2002 to 2013. Samples were preserved in ethanol (>95%), in RNAlater, or at -20°C prior to DNA extraction. DNA was extracted using the DNeasy Blood and Tissue Kit (Qiagen) or the Fast Tissue-to-PCR Kit (Fermentas, Thermo Scientific).

The species status of several preserved specimens was confirmed by mitochondrial gene (*COI* and *Cytb*) sequencing, either from previous work [5, 29] or, where new samples were available, by amplifying and sequencing the same regions (S2 Table). PCR amplification followed the protocols of Behere et al. [29] and Tay et al. [5]. PCR products were sequenced at Macrogen (Seoul, Korea) and the Biological Resources Facility (Australian National University, Canberra, Australia). Assembly of DNA trace sequences was performed using CLC Genomics Workbench version 8.0.

### Screening for *CYP337B3*

Following species identification, samples were screened for the presence of *CYP337B3* using the CYP337B2F and CYP337B1R primers described in Joussen et al. [18]. Heterozygote/homozygote status was determined through relevant band detection on 1.5–2% agarose gels containing 1% (w/v) of GelRed (Biotium) and visualised under UV light. Initial sequence was generated from these short fragments for a subset of samples following the PCR amplification protocol of Joussen et al. [18]. However, in order to generate more information, primers were designed to amplify the intron of the gene (S2 Table). In the chimeric *CYP337B3* gene, *CYP337B1* contributes the intron and several hundred base pairs of coding sequence depending on the allele, while *CYP337B2* contributes much of the 5' end of the coding sequence. Thus, the *CYP337B3* intron corresponds to the *CYP337B1* intronic sequence. We also screened samples from Brazil (n = 181) and Australia (n = 97) for the reciprocal recombination event using CYP337B1F and CYP337B2R primers (S2 Table) but no products were detected with this primer combination.

Further amplification of full-length genes and transcripts was performed for selected individuals with representative alleles. PCR products and cloned fragments were sequenced at several institutes, including: Macrogen (Seoul, Korea), Biological Resources Facility (Australian National University, Canberra, Australia), UC Davis Genome Center (University of California, Davis, US), StarSEQ (Mainz, Germany), Bioneer Corporation (Daejeon, Korea) and the Department of Entomology at the Max Planck Institute for Chemical Ecology (Jena, Germany). Assembly and sequence analysis of the PCR products was done using CLC genomics workbench v8.0 and T-Coffee (http://www.ebi.ac.uk/Tools/msa/tcoffee/).

### Resistance bioassays

To establish the role of *CYP337B3* in pythrethroid resistance, fenvalerate field resistant and susceptible individuals were identified by bioassay of field-collected material. Fenvalerate (95.3%) was provided by Sumitomo Chemical (Sydney, Australia) and dissolved in analytical grade acetone to produce a diagnostic concentration (0.125 μg/μL). A 1 μL volume of the discriminating concentration was applied to the dorsal thorax of late 3^rd^/early 4^th^ instar larvae within a weight range of 30-40mg using a 50 μL micro-syringe in a repeating dispenser (Hamilton Company, Reno, NV, USA). Bioassays were maintained for 7 d at 25°C with a 14:10 (L:D) h cycle. Relative humidity (RH) was not controlled. Dead and moribund larvae were collected and described as sensitive by using one or more of the following criteria: larvae unable to demonstrate coordinated movement when prodded; paralysis of prolegs; larvae very slow to right themselves (time exceeding 3 s). Larvae that were actively feeding and developing normally were described as resistant.

### Population genetic analysis

The elimination of variation in regions linked to an adaptive allele on one or even a few haplotypes is referred to as a “selective sweep” [30]. This process will lead to low nucleotide diversity (π) across the region of the genome under selection as well as an excess of rare variants as highlighted by Tajima’s D [31]. This statistic identifies deviation from a neutral model of evolution, whereby a negative score signifies purifying selection or population expansion that one would expect following a selection event. To provide evidence that the *CYP337B3* gene is under selection in *H*. *armigera*, we searched for genomic signals of a recent selection event using high-throughput sequencing data.

DNA samples from 12 Australian *H*. *armigera* homozygous for *CYP337B3v1* were sequenced on an Illumina HiSeq. Briefly, Nextera libraries were produced following the manufacturer’s instructions and sequence was generated as 100 bp PE reads (Illumina HiSeq 2000, Biological Resources Facility, Australian National University, Canberra, Australia). Raw reads were aligned to the bacterial artificial chromosome (BAC) 33J17 (JQ995292.1) sequence using BBMAP v33.43 (http://sourceforge.net/projects/bbmap/), trimming reads when quality in at least 2 bases fell below Q10. We did not align reads to the reference *H*. *armigera* genome sequence, which contains only *CYP337B1* and *CYP337B2* but not *CYP337B3* [32]. Only uniquely aligning reads were included in the analysis, to prevent spuriously inferring evolutionary processes occurring independently on each BAC. Outputted BAM files were sorted before duplicate reads were removed and files were annotated with read groups using Picard v1.138 (http://picard.sourceforge.net). The reference sequences were indexed using Samtools [33]. UnifiedGenotyper in GATK v3.3–0 [34] was used to estimate genotypes across all individuals simultaneously, implementing a heterozygosity value of 0.01. Genotypes annotated as “LowQual” were removed prior to subsequent analysis with VCFtools v0.1.12b [35], whereby π and Tajima’s D were calculated in sliding windows of 2500 bp that progressed by 1250 bp across biallelic sites of 33J17. Results were plotted in R v3.1.2 using ggplot2 v1.0.1 [36], while gene annotations were derived via tblastx [37] and visualised with CLC Genomics workbench v8.0.

To examine potential mechanisms underlying the origin of the *CYP337B3* alleles, an intron dataset containing sequences for representative *CYP337B1* and *CYP337B3* individuals (since the chimeric *CYP337B3* intron comes from the *CYP337B1* parental gene; see above) was created. MAFFT ver. 7.182 [38] was used to align these sequences using the -linsi option. Subsequently, IQ-TREE ver. 1.3.0 [39] was used, first in the -m TESTNEWONLY mode to determine the appropriate nucleotide substitution model, and then in full mode to generate a maximum likelihood tree. Subsequently 10,000 bootstrap replicates were carried out, and the bootstrap values transferred from the consensus tree to corresponding nodes of the maximum likelihood tree.

BEAST ver. 2.3.0 [40] was used for the intron-only dataset to test whether the *CYP337B3* allele is monophyletic in origin (i.e., has arisen once from a *CYP337B1*/*CYP337B2* cross-over and subsequently diverged independently). Two analyses were performed–one, in which monophyletic taxon sets for each of the *CYP337B1* and *CYP337B3* introns was enforced, and the other in which no monophyly was assumed. In BEAST, all runs used the evolutionary model identified in IQ-TREE (HKY + G4), a constant population coalescent tree prior, and a chain length of 10 x 10^6^. Other coalescent tree priors were also tested, but these did not quantitatively impact the results. BEAST results were examined in Tracer to confirm convergence and check ESS values (all exceeded 1,500). The likelihood of each analysis was then compared using Bayes Factors and the ACIM model, with 1,000 replicates, in Tracer ver. 1.6 [41].

## Results

A total of 1063 insect samples was collected from 18 countries around the world (Fig 1). The identification of *H*. *armigera* was initially based on morphological assessments by collectors and subsequently confirmed with the mitochondrial markers, *cytochrome oxidase I* and *cytochrome b* [29]. Any individuals not identified as *H*. *armigera* by mitochondrial DNA were excluded from subsequent analysis (*H*. *armigera* n = 999, *H*. *zea* n = 59, *Chloridea* (*Heliothis) virescens* n = 5).

**Fig 2:**
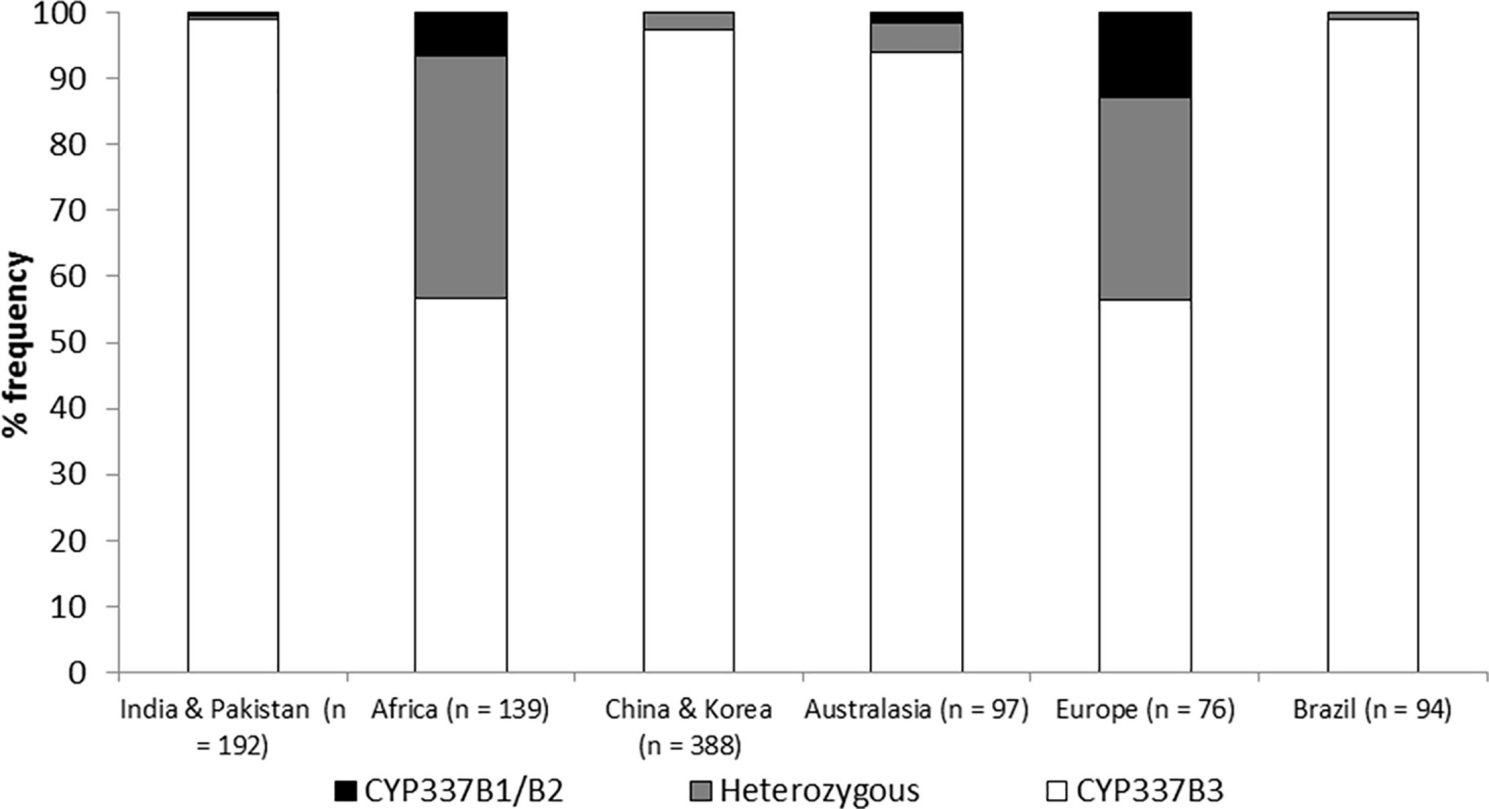
The frequency of *CYP337B* genotypes in selected geographic regions. For a more detailed breakdown see S1 Fig through S5 Fig.

A PCR assay using one primer from each of the two parental genes *CYP337B1* and *CYP337B2* was used to identify the presence of the chimeric *CYP337B3* gene. *CYP337B3* was extremely common in field collected *H*. *armigera* throughout its native range (Fig 2 and S1–S5 Figs). From a total of 999 individuals tested, *CYP337B3* was present in 969 (97%) and only 30 (3%) individuals were homozygous for *CYP337B1* or *CYP337B2*. 878 individuals were *CYP337B3* homozygotes (88%) and 91 were heterozygotes, meaning they carried one chromosome carrying *CYP337B3* and the other chromosome with the two parental genes *CYP337B1* and *CYP337B2* (9%). In some countries, almost all individuals were at least heterozygous for the *CYP337B3* gene, but homozygous *CYP337B3* individuals were the most common everywhere. In India, Pakistan, Korea, China, and Australia, almost all individuals were homozygous for *CYP337B3*, while heterozygotes (*CYP337B1*, *CYP337B2* and *CYP337B3*) at the *CYP337B3* locus were more common in European and African samples (Fig 2 and S1–S5 Figs).

**Fig 3:**
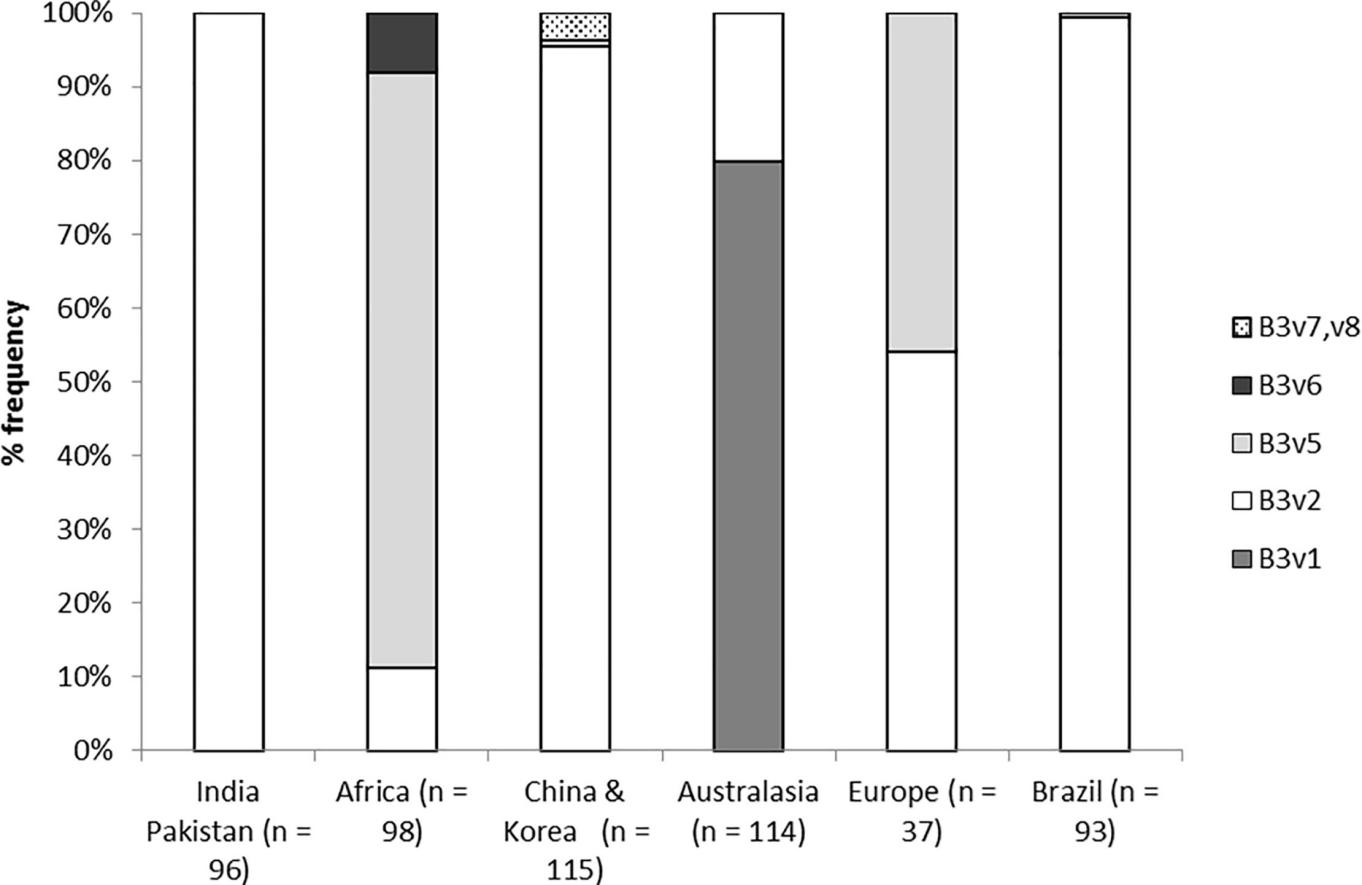
Frequencies of the different alleles of *CYP337B3 (*B3vX*)* in different geographic regions. Africa = Burkina Faso, Cameroon, Chad, Ghana, Madagascar, Senegal, Uganda; Australasia = Australia and New Zealand; Europe = Greece, France and Spain. n = the number of alleles sequenced, homozygotes and heterozygote alleles summed.

The PCR product of *CYP337B3* used in the screening PCR reaction [18] was sequenced from positive individuals (alleles n = 460) and a pattern of single nucleotide polymorphisms was identified. By comparing this short (355 bp) sequence between individuals, 6 alleles (*CYP337B3v1-2*, *5–8*) were identified worldwide (Fig 3). An allele originally identified in an Australian (Toowoomba) strain [18] was found only in Australia in the current study, where it was the most common allele (*v1*: n = 91), and *v2* the next most common allele (*v2*: n = 23). In Asian samples, *v2* was the most common allele (n = 203) and two other rare alleles were identified (*v7* and *v8*; n = 1 each) in addition to *v3* and *v4* identified by Han et al. [22]. Three alleles were found in Africa including two that were new (*v2*: n = 11, *v5*: n = 79, *v6*: n = 8). In the European samples, we identified an approximately 50:50 ratio of *CYP337B3v2* and *v5* (v2: n = 20; v5 n = 17). In addition, a proportion of samples from Australia (n = 11), Africa (n = 2), and Europe (n = 7) appeared from the sequencing traces to be heterozygous for different *CYP337B3* alleles. Overall, the various *CYP337B3* alleles appeared to show distinct patterns for each region, suggesting different alleles are present for pyrethroid resistance around the world.

**Fig 4:**
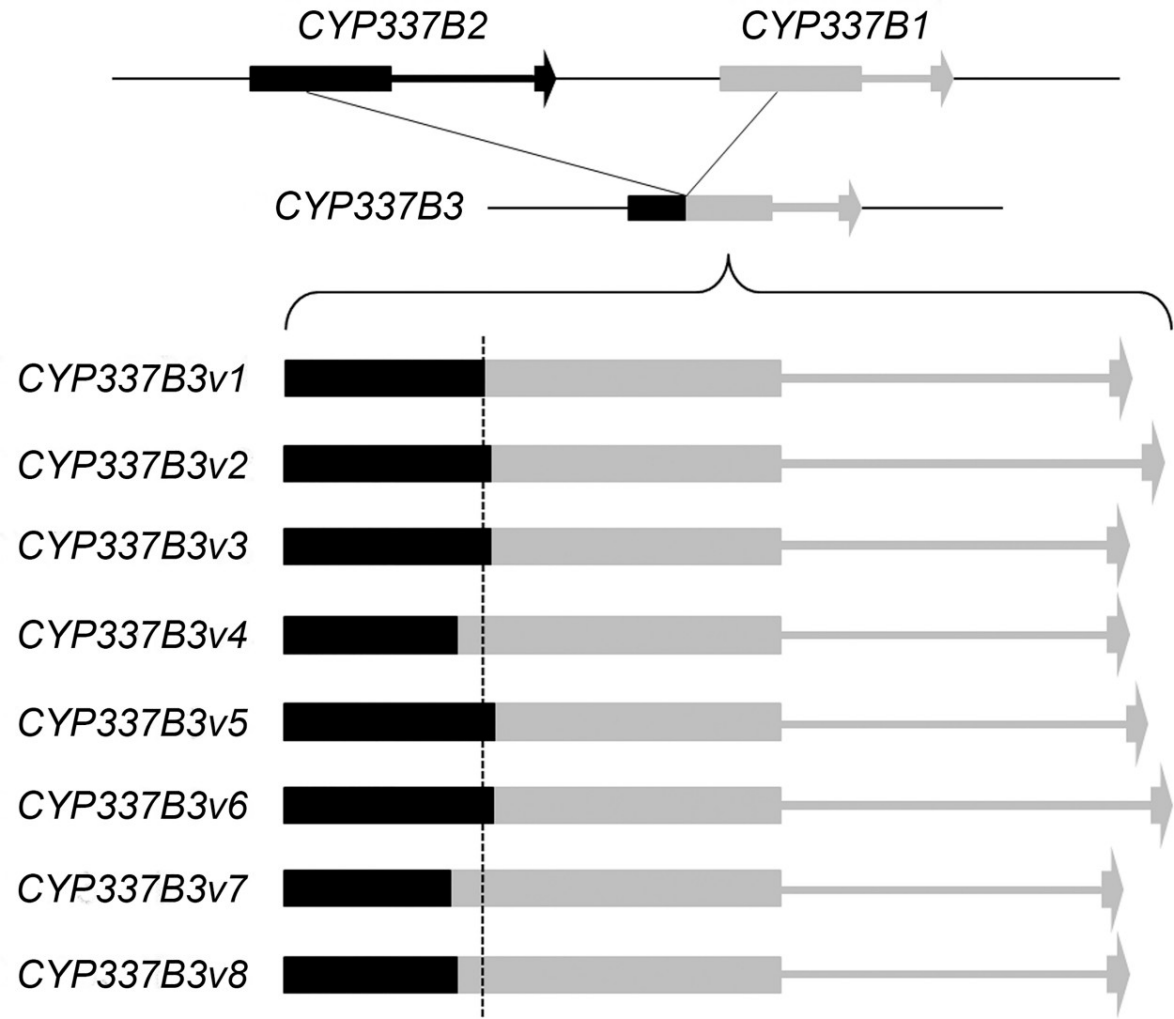
A schematic of the different crossing-over points and intron sizes observed in the chimeric *CYP337B3* gene.

Sequencing of the entire coding sequences of all variants revealed a number of non-synonymous SNPs resulting in different predicted proteins when compared to *CYP337B1v1*: 3 in *v2* [21], 3 in *v5*, 8 in *v6*, 9 in *v7*, and 11 in *v8*. All variants of the chimeric gene appear to show a similar pattern with slightly different cross over points between *CYP337B1* and *CYP337B2* during the formation of *CYP337B3* and different intron sequences and sizes that derived from *CYP337B1* determined by sequencing genomic DNA (Fig 4; S6 Fig; S3 Table). This suggests that multiple origins of the same functional phenotype from different genetic backgrounds define the development of this chimeric gene.

All of the 93 *H*. *armigera* individuals collected in Brazil were positive for *CYP337B3*, and all except one were homozygous. The majority of *CYP337B3* alleles detected in Brazilian *H*. *armigera* were identical to *CYP337B3v2*, which is predominantly found in Asia. However, the single heterozygous individual was carrying an African allele (*CYP337B3v5*). This suggests that Asia may be the most likely source for the Brazilian incursion of *H*. *armigera*, however, other source populations are also possible, as is the occurrence of multiple incursions. As well as examining *H*. *armigera*, we also examined *H*. *zea* from Brazil (n = 59) and we were able to amplify *CYP337B3* from one individual from Goias while from the others (n = 23) only *CYP337B1* provided consistent results. DNA from the suspect individual (S1 Table) was re-extracted twice in a different laboratory, using new reagents (extraction kit, primers, PCR reagents) and PCR was performed for the *CYP337B3* gene, intron and the mitochondrial marker COI. Extraction blanks were performed and were negative for both the COI sequence and the *CYP337B3*. Sequencing of both fragments from the three different extractions confirmed the presence of the *CYP337B3v2* allele (as found in the Brazilian *H*. *armigera*) in an individual with a mitochondrial sequence from *H*. *zea*.

Bioassays for pyrethroid resistance on *H*. *armigera* from cotton growing areas in eastern Australia using the discriminating dose clearly showed a higher frequency of homozygotes for *CYP337B3* in survivors (100% *CYP337B3* homozygous; n = 26) than in dead or dying insects (9 *CYP337B3* homozygotes, 2 heterozygotes and 1 homozygous *CYP337B1*/*B2* individual; n = 12), although a subset of the dead or dying caterpillars was actually positive for *CYP337B3*. In addition to bioassayed individuals, samples were collected from crops in Kununurra, Ord River Irrigation Scheme, Western Australia seven days after a field application of fenvalerate on cotton. Survivors were found to possess *CYP337B3*, with a 100% homozygous frequency (n = 7). Insects collected from the same region on unsprayed crops (cotton, chickpea and chia) showed a lower frequency of *CYP337B3* (2 homozygote *CYP337B3*, 1 heterozygote and 3 homozygote *CYP337B1*/*B2* individuals; n = 6). Although these very small samples from field populations are insufficient for claims of statistical significance, they are consistent with the significant genotypic differences previously found in the Toowoomba laboratory population derived from Australia [18].

**Fig 5:**
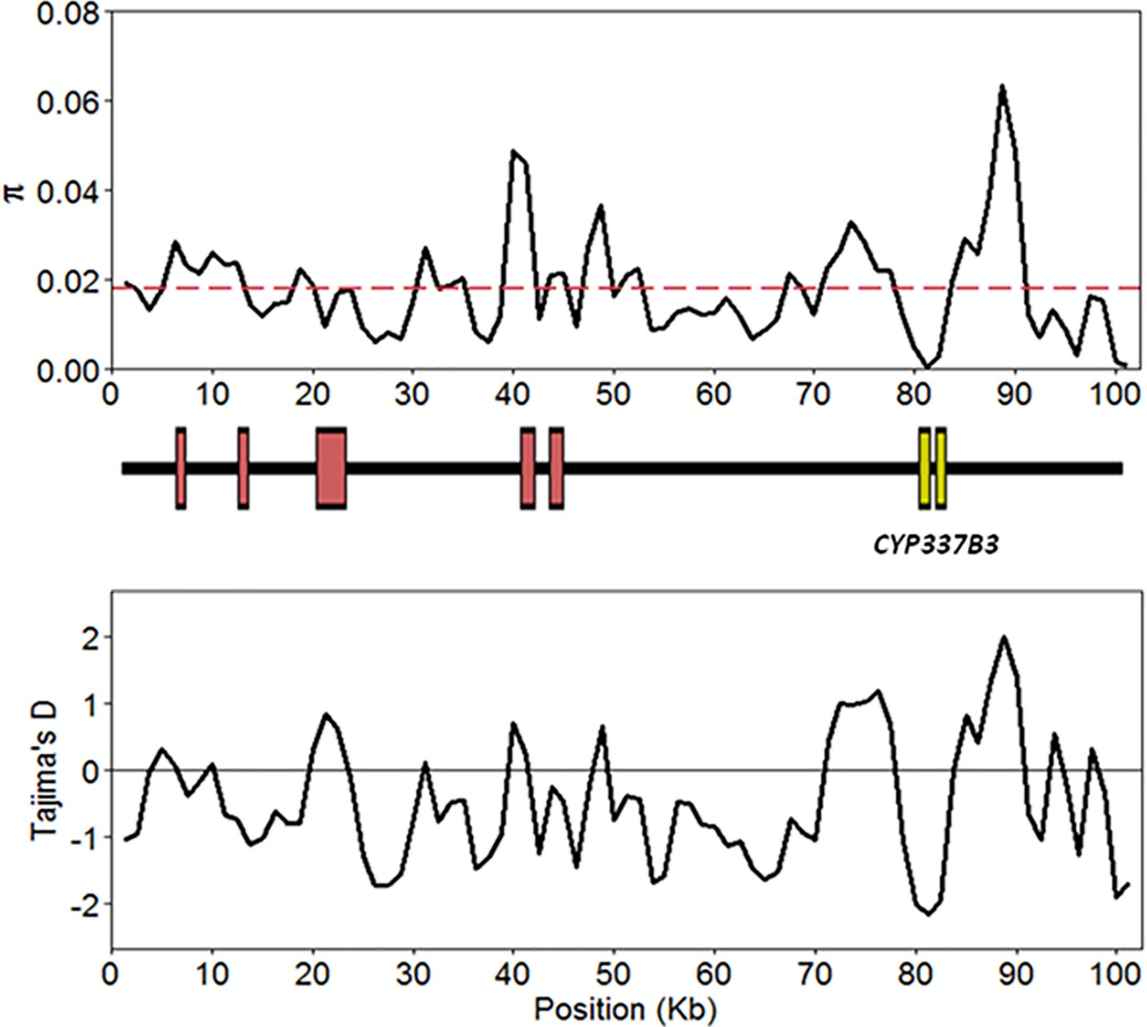
Selective sweep among Australian *H*. *armigera* homozygous for *CYP337B3v1*. (a) Nucleotide diversity (π) and (b) Tajima’s D calculated across sliding windows of the 33J17 BAC (JQ995292.1), with the red dashed line showing the average value of π (0.0179). Locations of gene bodies are indicated between the plots, with those in red identified as potential reverse transcriptases and those in yellow as exons of *CYP337B3v1*.

Using individuals from Australia that were homozygous for the *CYP337B3v1* allele, high throughput sequencing data was aligned to the BAC clone *33J17* which was the original source of the *CYP337B3v1* identification and originates from the Australian Toowoomba strain [18]. Of the 100,377 genotypes called across *33J17*, 32,381 remained after “LowQual” SNPs were removed. Both π and Tajima’s D were variable, however values were decisively differentiated where *CYP337B3* lies; nucleotide diversity reaches its lowest point at the *CYP337B3* locus (0.00057) relative to the BAC-wide mean at 0.0179 (Fig 5; S4 Table). This is the expectation following a “hard” selective sweep, whereby adaptive alleles appear as a single haplotype before increasing in frequency [30]. Tajima’s D is also lowest across the region where *CYP337B3* is located (-2.18) and points towards the process of purifying selection. It is worth noting that the BAC-wide mean value for Tajima’s D is -0.43; such a low negative value may signify that other evolutionary processes have also acted upon this region [42]. While this variability is likely a result of small sample sizes and relatively small sliding window values, the small size (~ 5 kbp) of the region around *CYP337B3v1* may signify ongoing high recombination rates and gene flow [43, 44].

To establish the potential mechanisms that underlie the creation of the chimeric gene and account for its global distribution, we sequenced the intron of *CYP337B3*. This region should contain more polymorphisms and allow a better diagnosis of the frequency of the different genotypes. As for the coding region data (above), we found a pattern of eight alleles in the intron data. Introns sequenced from *B3v3*, *v4* and *v8* are very similar and differ only by two SNPS whereas introns from *B3v1*, *v2*, *v5*, *v6* and *v7* show more significant differences in both length and sequence (Fig 4). Individuals that were homozygous for the *CYP337B3* gene were predominantly homozygous at the allele level, but as in the coding region, heterozygotes were again observed in the sequence traces of a small number of samples from Africa, Australia and Europe. Cloning and sequencing of the heterozygous PCR products showed that, in Australia, *v1/v2* (Australia/Asia) heterozygotes were present, while in Africa, there were heterozygotes of *v2/v5* (Asia/Africa) and *v5/v6* (both African). European heterozygotes possessed *v2/v5* (Asia/Africa).

Phylogenetic relationships were inferred for the intronic region of a subset of representative *CYP337B1* and *CYP337B3* sequences. If the chimeric *CYP337B3* were due to a single cross-over event followed by subsequent divergence of allelic variations, then the phylogenetic estimates should show a monophyletic clade with the *CYP337B3* clade nested within the *CYP337B1 clade*. Our tests with BEAST indicated that this was not the case; specifically Bayes Factor (BF) analysis found overwhelming support for a paraphyletic origin of *CYP337B3* (BF score for paraphyly *vs*. monophyly = 494.412, with BF > 10 indicating very strong evidence against the null model of monophyly). This does not rule out the possibility of some subsequent recombination between derived *CYP337B3* alleles and either parent gene.

**Fig 6:**
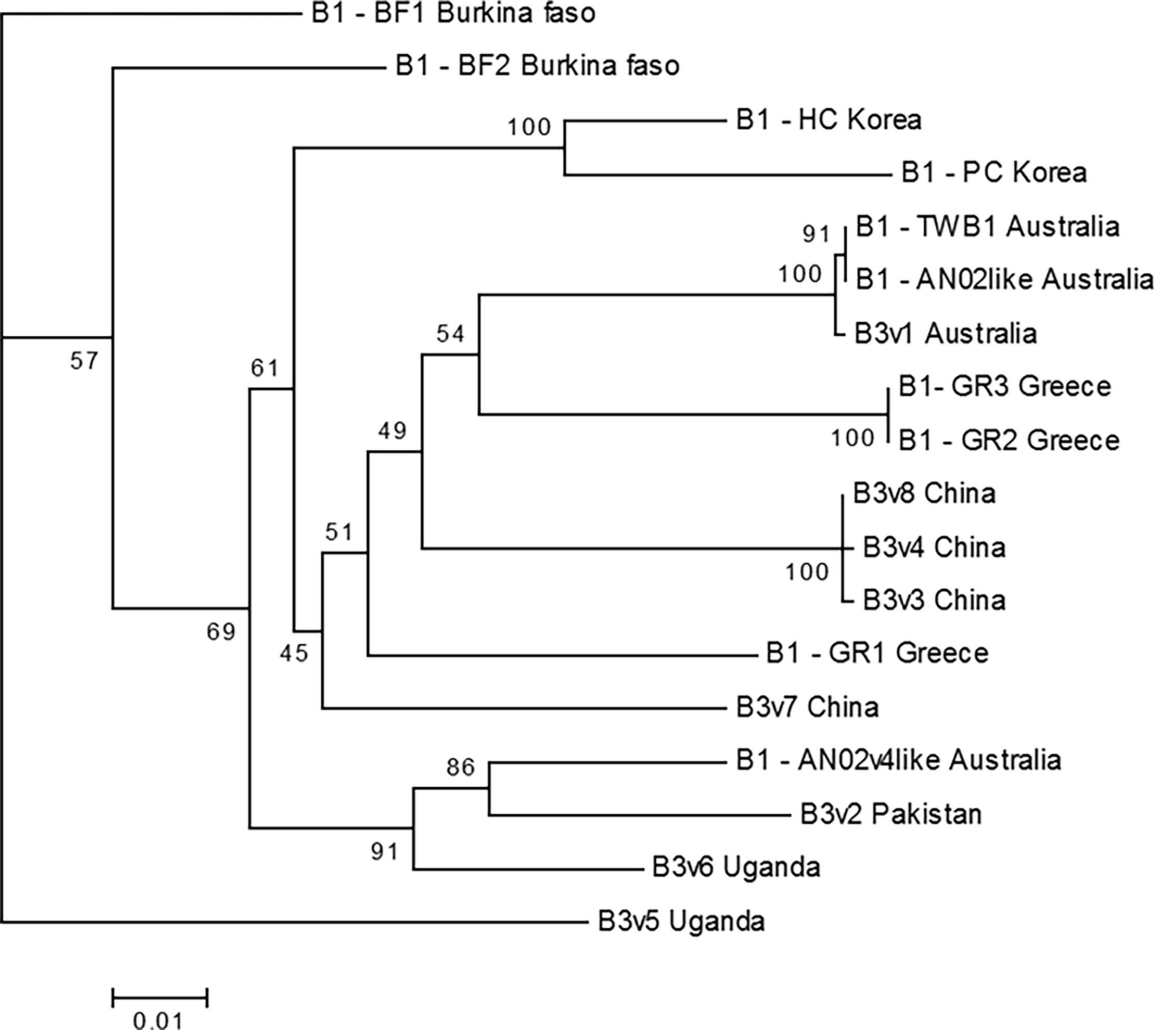
Maximum likelihood phylogeny of CYP337B1 and CYP337B3 alleles. The maximum likelihood tree was generated in IQ-TREE with the HKY+G4 model of sequence evolution, using representative *CYP337B1* and *CYP337B3* intron sequences. Bootstrap values from 10,000 replicates are given on tree nodes. The eight *CYP337B3* alleles are indicated with labels (v1:v8), different CYP337B1 alleles are indicated by B1- followed by an allele ID and the country of origin. Sequences and related NCBI identifiers can be found in S3 Table and S1 File.

Fig 6 presents the results of the maximum likelihood phylogenetic analysis, which also suggests multiple origins of the various *CYP337B3* alleles. Although bootstrap support values for some nodes of the tree are quite low, several well-supported relationships are present, and these are consistent with multiple geographic origins for the *CYP337B3* alleles. For example, *CYP337B3v1* (found only in Australia) is most closely related at the sequence level to a *CYP337B1v1* allele from Australia. Furthermore there is a cluster of closely related but rare *CYP337B3* alleles found in China (*CYP337B3v3*, *v4*, and *v7*) which could indicate local origins for those alleles. Thus, while some *CYP337B3* alleles may be recently derived from local *CYP337B1* alleles, the overall signal indicates that multiple unequal crossing-over events are responsible for the geographic distribution of chimeric *CYP337B3* while gene flow as well as some intergenic recombination may also have played a role producing the current geographic distribution patterns of *CYP337B3* alleles.

We performed an additional calculation to determine the molecular rate that would be required to produce the measured *CYP337B3* allelic diversity if it had all arisen in the last ~50 years. Even using the rapidly evolving mitochondrial divergence rate of 1.5–2.5% divergence per million years as a baseline [45], we found that a 1000-fold increase in this rate would be required to place all *CYP337B3* divergence events inside a 50-year time window. This rate is so high, it suggests that rather than recently arising through *de novo* mutation in response to pyrethroid applications and then spreading around the world, cytochrome P450-mediated pyrethroid resistance is most likely the result of selection acting over the last 50 years on existing genetic variation present in the populations at low frequency.

## Discussion

*Helicoverpa armigera* is one of the world’s most destructive pests and has developed resistance to numerous pesticides over the years [27]. Globally, pyrethroids are one of the most commonly used insecticides and resistance occurred rapidly at a global scale after the first use of pyrethroid insecticides in the late 1970s [27]. A number of mechanisms have been proposed for this resistance, including target site insensitivity [27, 46, 47], and metabolism by carboxylesterases [48, 49] and P450s [50–56]. Recent work suggests that a chimeric gene, *CYP337B3*, may play an important role in cytochrome P450-mediated pyrethroid resistance and we set out to examine the frequency of this gene worldwide [18, 21].

We detected the chimeric *CYP337B3* gene in every population examined and found a total of eight allelic variants of *CYP337B3* globally. Of the eight global alleles identified, one was found only in Australia (*CYP337B3v1*), one was dominant across Asia (India, Pakistan, China, Korea), and Brazil (*CYP337B3v2*), four were rare and uniquely found in China (*CYP337B3v3*, *v4*, *v7 and v8*) and two were predominantly African (*CYP337B3v5*, *v6*). European samples showed a mixture of alleles (*CYP337B3v2*, *v5*). *CYP337B3v2* was the most widespread allele, identified on every continent examined but was present at the highest frequencies in Asia and Brazil.

One issue in using *CYP337B3* as a population marker is that diversity in *CYP337B3* is not new diversity, but rather a re-shuffling of diversity already present in the parental *CYP337B1* and *CYP337B2* genes. However, the presence of multiple alleles at the *CYP337B3* locus suggests that this chimeric gene has formed independently several times over the evolutionary history of the locus. In the overall patterns, we found that three alleles, *v1*, *v2* and *v5*, together account for ~98% of all detected *CYP337B3* alleles. The geographic distribution of these common alleles may suggest older recombination events in Australia, Asia, and Africa producing the common alleles and potentially more recent additional events in Asia and Africa producing the rare alleles. The alternative explanation that a single event has occurred, and then been distributed globally by migration and subjected to subsequent local recombination, is much less likely as it is not supported by the phylogeny or the molecular rate analysis. Indeed, our phylogenetic analysis found strong support for a paraphyletic origin of *CYP337B3* rather than the alternative of a single unequal cross-over event followed by subsequent allelic divergence. In addition, our calculations suggest that a 1000-fold increase in a commonly accepted baseline divergence rate (1.5–2.5% divergence per million years) would be required to place all *CYP337B3* divergence events inside a 50-year time window [45]. Taken together, the allele frequencies and phylogenetic analysis suggest that, rather than recently arising through *de novo* mutations in response to pyrethroid application, cytochrome P450-mediated pyrethroid resistance is most likely the result of recent selection acting on existing genetic variation maintained in the population at low frequency. Our selective sweep analysis supports this scenario, with patterns of estimated π and Tajima’s D across the *33J17* BAC indicating that the *CYP337B3* locus has undergone recent selection, at least in Australia and with regard to *CYP337B3v1*. Recent work confirms that this can also be shown for the other alleles [26]. Further work will reveal how the diversity in this one region compares to the rest of the genome.

There are a number of examples in the literature where the same insecticide resistance mechanisms have developed independently in different locations. In particular pyrethroid resistance (perhaps because of the short period between the introduction and the development of resistance) associated with identical point mutations in the voltage-gated sodium channel has developed in different haplotypes with different geographical origins in mosquitoes and houseflies [57, 58]. In the sheep blowfly, the same mutations in an esterase gene conferring organophosphate resistance were identified in very different haplotypes [59].

Although further work incorporating whole genome scale markers will be necessary to determine the origin of *H*. *armigera* in the New World and global patterns of movement in this species [26, 28], we gain insight into some of these processes here. The vast majority of the Brazilian insects examined possessed the allele *CYP337B3v2* found predominantly in Asia. However, we also detected the *CYP337B3v2* allele in other regions of the world, and so it is possible that the Brazilian populations of *H*. *armigera* could have come from localities where the *CYP337B3v2* allele also exists. For example, the closest native population of *H*. *armigera* to the New World is in West Africa and it is possible that the New World *H*. *armigera* are the result of a similar incursion from West Africa. However, only Asia has close to the same proportion of *CYP337B3v2* alleles. A recent survey [8] showed that the pattern of mitochondrial markers in *H*. *armigera* populations collected from southern Brazil, Argentina, Uruguay and Paraguay is very different from *H*. *armigera* populations from central and northern regions of Brazil, suggesting multiple incursions of this global pest into the New World [60]. It would be interesting to examine the *CYP337B3* locus in these individuals to see if they share the same pattern as found in Brazil.

Recently, evidence for other pyrethroid detoxicative mechanisms in addition to *CYP337B3* has been found in China [22], and our own data showed some mortality in *CYP337B3-* homozygous individuals. This might suggest that possession of the *CYP337B3* is not enough for resistance alone and other mechanisms related to expression or other genes may exist. However, as the resistance based on *CYP337B3* is metabolic, it is not unusual that a high dose of insecticide will kill even homozygous individuals, for example if the enzyme is not abundant enough to decrease the amount of insecticide at its target site beneath the toxic threshold. Furthermore, we clearly see evidence of conservation of the *CYP337B3* alleles at this locus across the globe and we would not expect the observed level of conservation in the absence of selection, particularly in the non-coding regions. Indeed, when we examined individuals from Australia we found evidence of a selective sweep around the gene, indicating maintenance of the allele at this locus. Throughout its native range, phenotypic pyrethroid resistance is very common in *H*. *armigera* and it is of course possible that other or additive resistance mechanisms are at work in populations around the world.

The arrival of *H*. *armigera* in the New World represents a significant expansion of its geographc range. It remains to be seen how large the problem *H*. *armigera* will become but, clearly, arriving with a resistance gene already in place would provide *H*. *armigera* a selective advantage over local susceptible populations of pests such as *H*. *zea* and *Chloridea* (*Heliothis*) *virescens*. One further complication is that *H*. *zea* has been shown to interbreed with *H*. *armigera* in the laboratory to produce viable offspring [61–63] and there is evidence of hybridisation in Brazil using genome wide markers [28]. In the current study, we applied the same PCR reaction conditions to individuals identified as *H*. *zea* from Brazil and we able to amplify *CYP337B3v2* from one individual. Clearly this one result is not enough to suggest that hybridisation is widespread between the two species but the detection of a presumably highly selectable gene indicates that this is something that should be considered a risk. While it is unclear what risk hybrids may pose, it is certain that an exchange of resistance and host association genes could lead to significant problems in control. The fact that the presence of the chimeric resistance gene *CYP337B3* can be ascertained by a simple screening PCR enables the rapid determination of susceptible *vs*. pyrethroid-resistant populations, which could lead to improved pest management strategies and the detection of hybrid individuals in the field.

This work highlights a case of resistance alleles in *H*. *armigera* evolving on a local scale in a global context and provides an example of a species invading new continents, already able to overcome some of the control measures deployed for other species. We also show that resistance genes can arise locally and then mix on a global scale as a result of either natural movement or human-mediated transport. The implications for *H*. *armigera* and potentially *H*. *zea* as species are unclear but what is not in doubt is that the pesticide resistance selection pressure on *H*. *armigera*, in terms of numbers exposed, has dramatically increased with this incursion. This is true not just for conventional pesticide resistance but also for transgenic insecticidal traits. Transgenic cotton and especially corn and soybean which express Bt toxins are grown over a far larger area in the New World and the management of Bt and conventional pesticide resistance should be a priority.

Ultimately, we grow the same crops around the world, control pests using the same chemistries, and transport commodities through global trade routes, so it is not a surprise that we are developing global, well-adapted pests. Our paper not only sets the scene for further work in this area but also highlights the value of considering biosecurity threats in terms of both the actual pest as well as its resistance profile (both phenotype and genotype).

## Supporting information

S1 Fig The frequency of *CYP337B3* on the Asian Sub-continent by region. Northern, Central and Southern represent general geographic regions of India.
(TIF)

S2 Fig The frequency of *CYP337B3* in selected African countries. Homozygous and heterozygous frequencies are shown.
(TIF)

S3 Fig The frequency of *CYP337B3* in China and Korea. *Larvae from lab colonies derived from each field collection (20–50 individuals) and maintained at a population size of 300–500 adults per generation without exposure to any insecticide for 3–5 generations.
(TIF)

S4 Fig The frequency of *CYP337B3* in Australia and New Zealand. Homozygous and heterozygous frequencies are shown.
(TIF)

S5 Fig The frequency of *CYP337B3* in Europe. Homozygous and heterozygous frequencies are shown.
(TIF)

S6 Fig Alignment of the crossover points for the various *CYP337B3* alleles. *CYP337B1* is in red, *CYP337B2* is green and the boxed sequence represents crossover point for each *CYP337B3* allele.
(TIF)

S1 Table Collection sites of *H. armigera* and *H. zea* from 18 different countries. (DOCX)

S2 Table PCR primers used in this work. (DOCX)

S3 Table Sequence identifiers, gene names, origin and NCBI numbers used for the maximum likelihood estimate of phylogenetic relationships. (DOCX)

S4 Table Sequencing and alignment statistics for the 12 individuals used to demonstrate the selective sweep around *CYP337B3v1*. (DOCX)

S1 File Intron sequence data used for the maximum likelihood estimate of phylogenetic relationships. (DOCX)
