# Supplementary figures and images for: Multiple recombination events between two cytochrome P450 loci contribute to global pyrethroid resistance in Helicoverpa armigera
Source: PLoS One. 2018 Nov 1;13(11):e0197760. doi: 10.1371/journal.pone.0197760 (PMC6211633; doi:10.1371/journal.pone.0197760)

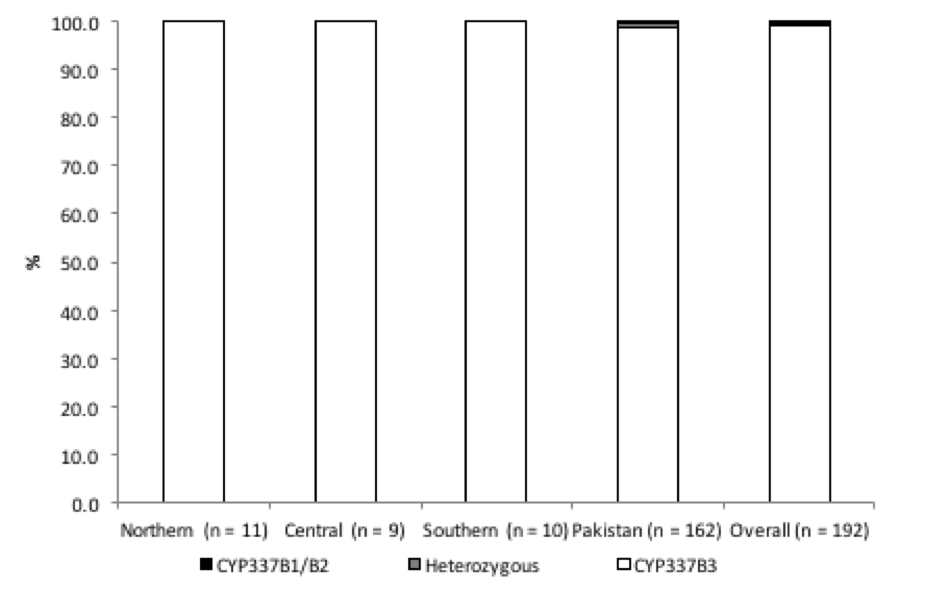

Supplement: S1 Fig — Northern, Central and Southern represent general geographic regions of India. (TIF) [file pone.0197760.s001.tif]

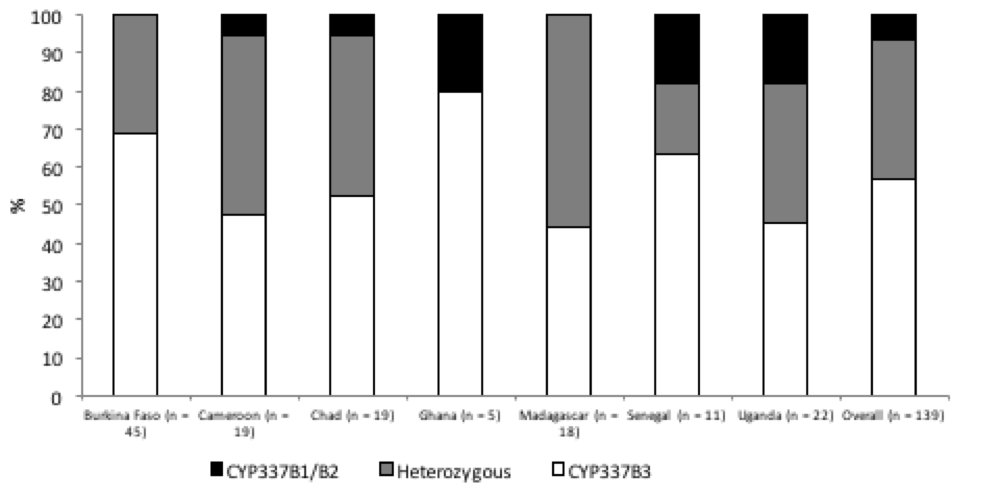

Supplement: S2 Fig — Homozygous and heterozygous frequencies are shown. (TIF) [file pone.0197760.s002.tif]

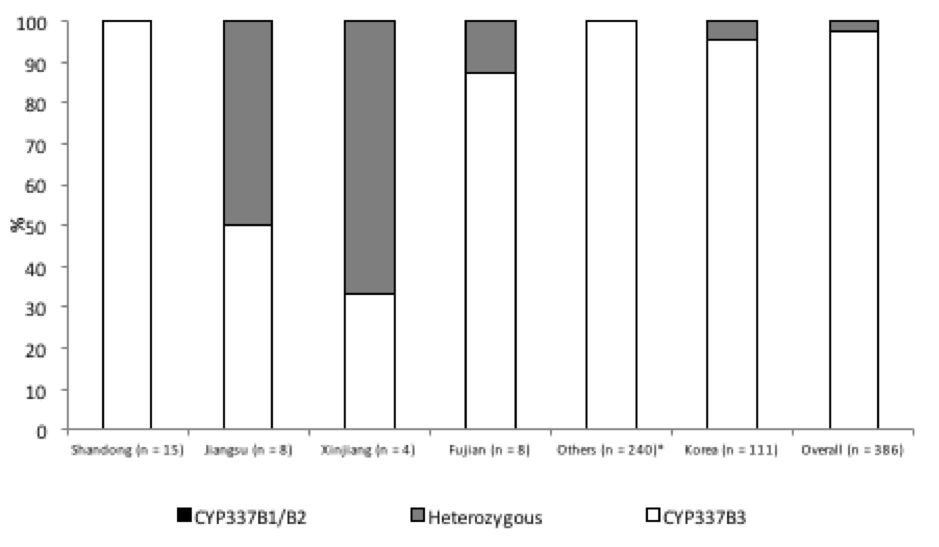

Supplement: S3 Fig — *Larvae from lab colonies derived from each field collection (20–50 individuals) and maintained at a population size of 300–500 adults per generation without exposure to any insecticide for 3–5 generations. (TIF) [file pone.0197760.s003.tif]

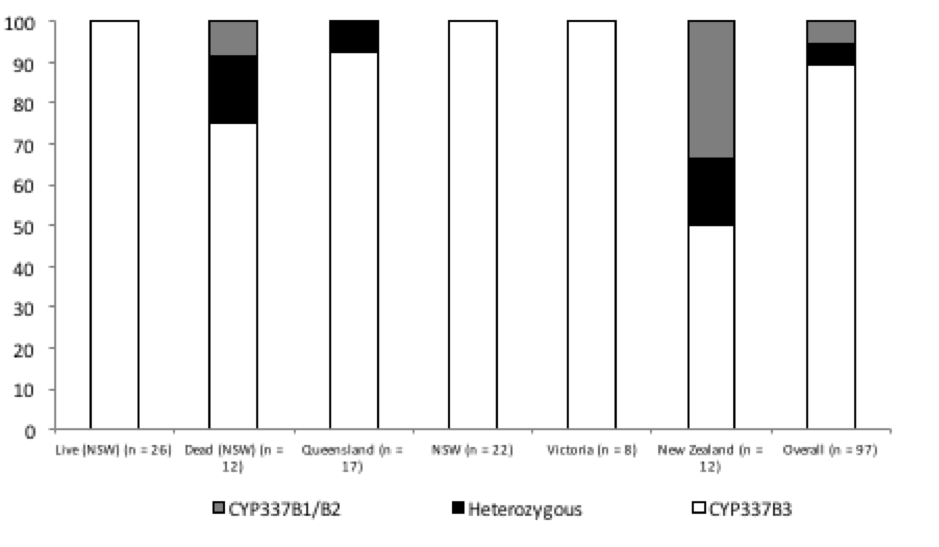

Supplement: S4 Fig — Homozygous and heterozygous frequencies are shown. (TIF) [file pone.0197760.s004.tif]

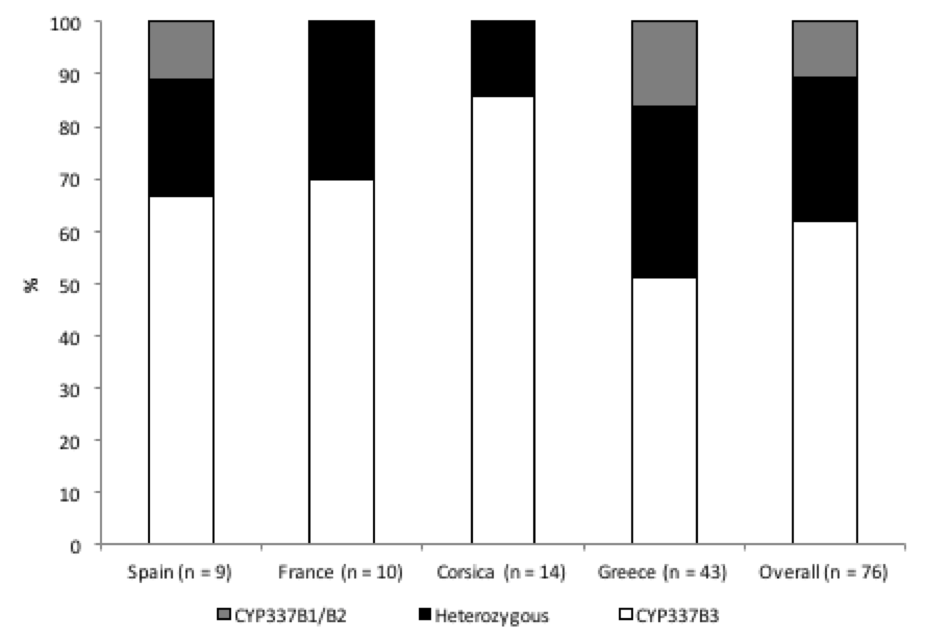

Supplement: S5 Fig — Homozygous and heterozygous frequencies are shown. (TIF) [file pone.0197760.s005.tif]

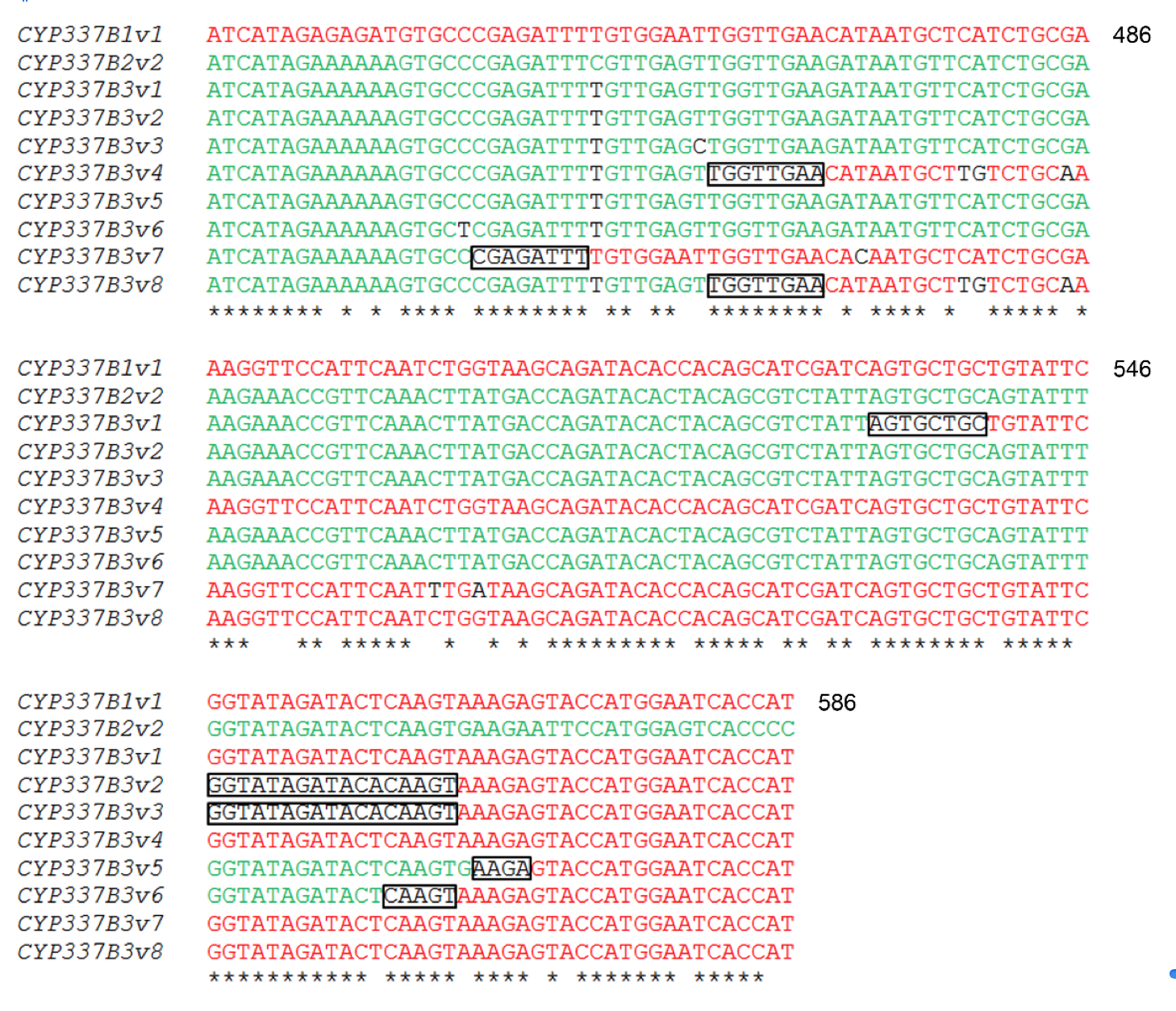

Supplement: S6 Fig — CYP337B1 is in red, CYP337B2 is green and the boxed sequence represents crossover point for each CYP337B3 allele. (TIF) [file pone.0197760.s006.tif]
