# Supplementary material for: Multiple recombination events between two cytochrome P450 loci contribute to global pyrethroid resistance in Helicoverpa armigera
Source: PLoS One. 2018 Nov 1;13(11):e0197760. doi: 10.1371/journal.pone.0197760 (PMC6211633; doi:10.1371/journal.pone.0197760)
Supplement: S1 Table — (DOCX) [file pone.0197760.s007.docx]

**S1 Table. Collection sites of *H. armigera* and *H. zea* from 18 different countries**

| Country | Region | Collection date | Stage | Lat. | Long. | Trap/Plant | No. |
| --- | --- | --- | --- | --- | --- | --- | --- |
| Greece | Larissa, Thessaly Periphery | Summer 2012 | Larvae | 39.62 | 22.60 | Cotton | 43 |
|  |  |  |  |  |  |  |  |
| Spain | Seville | 2013 | Adult | NA | NA | Alfalfa | 9 |
|  |  |  |  |  |  |  |  |
| France | Mainland | 2013 | Adult | NA | NA | NA | 10 |
|  | Corsica | 2013 | Adult | NA | NA | NA | 14 |
|  |  |  |  |  |  | **Total** | **24** |
| Pakistan |  |  |  |  |  |  |  |
|  | Gilgit, Gilgit-Baltistan | 20-Jun-13 | Adult | 35.92 | 74.29 | Light trap | 1 |
|  | Dassu, Gilgit-Baltistan | 19-Jun-13 | Adult | 35.27 | 73.22 | Light trap | 3 |
|  | Kel, AJK | 15-Jul-12 | Adult | 34.82 | 74.55 | Light trap | 1 |
|  | Peer Chinassi, AJK | 14-Jul-12 | Adult | 34.38 | 73.55 | Light trap | 2 |
|  | Ath Maqam, AJK | 17-May-12 | Adult | 34.59 | 73.91 | Light trap | 2 |
|  | Rawalakot, AJK | 9-Jun-11 | Adult | 33.85 | 73.74 | Light trap | 4 |
|  | Ghari Dupatta, AJK | 3-Oct-10 | Adult | 34.22 | 73.61 | Light trap | 1 |
|  | Kalam, Swat, KPK | 2-Jul-10 | Adult | 35.46 | 72.58 | Blackberry | 35 |
|  | Manglor, Swat, KPK | 1-Sep-12 | Adult | 34.77 | 72.46 | Light trap | 1 |
|  | Matta, Swat KPK | 11-Sep-12 | Larvae | 34.87 | 72.41 | Okra | 5 |
|  | Dargai, Malakand, KPK | 1-Sep-12 | Larvae | 34.51 | 71.92 | Okra | 5 |
|  | Charsadda, KPK | 16-Jun-13 | Larvae | 34.34 | 71.79 | Okra | 5 |
|  | Besham, KPK | 15-Jun-13 | Adult | 33.93 | 72.87 | Light trap | 1 |
|  | Balakot, KPK | 17-Jul-13 | Adult | 34.54 | 73.34 | Light trap | 2 |
|  | Shogran, KPK | 19-Jul-13 | Adult | 34.63 | 73.45 | Light trap | 2 |
|  | Mansehra, KPK | 20-Jul-10 | Adult | 34.32 | 73.2 | Light trap | 1 |
|  | Changla Gali, KPK | 5-Jul-12 | Adult | 33.98 | 73.39 | Light trap | 2 |
|  | Muree, Punjab | 16-Jul-13 | Adult | 33.9 | 73.39 | Light trap | 2 |
|  | Islamabad (Capital) | 26-Jul-10 | Adult | 33.71 | 73.05 | Light trap | 1 |
|  | Khewra, Punjab | 16-Apr-12 | Adult | 32.6 | 72.99 | Light trap | 5 |
|  | Jehlum, Punjab | 17-Aug-12 | Adult | 32.92 | 73.76 | Light trap | 2 |
|  | Chiniot, Punjab | 20-Apr-10 | Adult | 31.75 | 72.94 | Okra | 3 |
|  | Faisalabad, Punjab | 25-May-2009 to July 2014 | Adult | 31.39 | 73.03 | Chickpea/Alfalfa | 20 |
|  | Sheikhupura, Punjab | April 4, 2010 | Larvae | 31.74 | 73.99 | Alfalfa | 2 |
|  | Nankana Sahib, Punjab | April 4, 2010 | Larvae | 31.44 | 73.68 | Alfalfa | 2 |
|  | Changa Manga, Lahore Punjab | 13-Jun-12 | Larvae | 31.08 | 73.99 | Light trap | 2 |
|  | DG Khan, Punjab | 15-Apr-13 | Larvae | 30.93 | 70.65 | Chickpea | 5 |
|  | Mankara, Bhakkar Punjab | 25-Mar-13 | Larvae | 31.38 | 71.43 | Chickpea | 5 |
|  | Multan, Punjab | 25-May-09 | Adult | 30.13 | 71.44 | Light trap | 19 |
|  | Bahawalpur, Punjab | 8-May-12 | Adult | 29.35 | 71.67 | Light trap | 5 |
|  | Sadiqabad, Punjab | 9-May-12 | Adult | 28.29 | 70.1 | Light trap | 5 |
|  | Sanghar, Sindh | 25-Aug-10 | Adult | 26.03 | 68.93 | Light trap | 2 |
|  | Hatango Khupru, Sindh | 27-Mar-12 | Adult | 25.85 | 69.37 | Light trap | 2 |
|  | Nagar Parkar, Sindh | 7-Apr-12 | Adult | 24.35 | 70.76 | Light trap | 2 |
|  | Jamrao, Sindh | 18-Apr-12 | Adult | 25.41 | 68.98 | Light trap | 1 |
|  | Karchat, Sindh | 4-Apr-13 | Adult | 25.69 | 67.52 | Light trap | 3 |
|  | Mithi, Sindh | 10-Apr-12 | Adult | 24.73 | 69.78 | Light trap | 1 |
|  |  |  |  |  |  | **Total** | **162** |
|  |  |  |  |  |  |  |  |
| India | Nagpur | 2005 | Adult | 21.14 | 79.1 | Pheromone | 4 |
|  | Warangal | 2005 | Larvae | 17.99 | 79.64 | Cotton | 1 |
|  | Yavtamal | 2005 | Larvae | 20.39 | 78.12 | Eggplant | 2 |
|  | Coimbatore | 2005 | Larvae | 11 | 76.96 | Pigeon pea | 4 |
|  | Guntur | 2005 | Larvae | 16.36 | 80.44 | Cotton | 2 |
|  | Deli | 2005 | Larvae | 20.63 | 77.18 | NA | 1 |
|  | Southern India | 2005 | Larvae | NA | NA | Pheromone | 5 |
|  | Northern India | 2005 | Larvae | NA | NA | NA | 11 |
|  |  |  |  |  |  | **Total** | **30** |
|  |  |  |  |  |  |  |  |
| China | Nanping, Fujian |  | Adult | NA | NA | NA | 8 |
|  | Yancheng, Jiangsu |  | Adult | NA | NA | NA | 8 |
|  | Xiajin, Shandong |  | Adult | NA | NA | NA | 15 |
|  | Shache, Xianjiang |  | Adult | NA | NA | NA | 4 |
|  |  |  |  |  |  |  |  |
|  | Huanggang, Hubei | Jul-13 | Larvae (36) | 30.45 | 114.91 | Cotton | 30 |
|  | Qianjiang, Hubei | Jul-13 | Larvae (20) | 30.4 | 112.9 | Cotton | 30 |
|  | Anxiang, Hunan | Jul-13 | Larvae (30) | 29.41 | 112.17 | Cotton | 30 |
|  | Luoyang, Henan | Aug-13 | Larvae (28) | 34.67 | 112.44 | Cotton | 30 |
|  | Xuzhou, Jiangsu | Jul-13 | Larvae (34) | 34.27 | 117.17 | Cotton | 30 |
|  | Changdao, Shandong | Jul-13 | Adult (50) | 37.91 | 120.7 | Immigrant | 30 |
|  | Qihe, Shandong | Aug-13 | Larvae (23) | 36.8 | 116.77 | Cotton | 30 |
|  | Haimen, Zhejiang | Aug-13 | Larvae (40) | 31.89 | 121.17 | Cotton | 30 |
|  |  |  |  |  |  | **Total** | **275** |
|  |  |  |  |  |  |  |  |
| Korea | Suwon, Gyeonggi-do | 6-Sep-12 | Larvae | 37.26 | 126.97 | Tomato | 41 |
|  | Pyeongchang, Gangwon-do | 12-Aug-13 | Adult | 37.68 | 128.73 | Corn | 50 |
|  | Hongcheon, Gangwon-do | 12-Aug-13 | Adult | 37.70 | 127.86 | Corn | 17 |
|  | Jeju, Jeju-do | 7-Aug-13 | Adult | 33.47 | 126.52 | Pheromone | 5 |
|  |  |  |  |  |  | **Total** | **113** |
|  |  |  |  |  |  |  |  |
| Burkina Faso | Ouagadougou Centre | 2013 | Larvae | NA | NA | NA | 39 |
|  | Diapaga | 2002 | Larvae | NA | NA | Cotton | 6 |
|  |  |  |  |  |  | **Total** | **45** |
|  |  |  |  |  |  |  |  |
| Chad | Pala | 2006 | Larvae | NA | NA | Cotton | 19 |
|  |  |  |  |  |  |  |  |
| Cameroon | Bertoua | 2005 | Larvae | NA | NA | Maize | 19 |
|  |  |  |  |  |  |  |  |
| Ghana | Tamale | 2012 | Larvae | NA | NA | NA | 5 |
|  |  |  |  |  |  |  |  |
| Madagascar | Antsirabe | 2006 | Larvae | NA | NA | Maize | 18 |
|  |  |  |  |  |  |  |  |
| Senegal | Noto | 2005 | Larvae | NA | NA | Tomato | 11 |
|  |  |  |  |  |  |  |  |
| Uganda | Kampala | 2006 | Larvae | NA | NA | NA | 22 |
|  |  |  |  |  |  |  |  |
| Australia | New South Wales | 2012 | Larvae | NA | NA | NA | 62 |
|  | Queensland | 2012 | Adults | NA | NA | NA | 17 |
|  | Victoria | 2006 | Larvae | NA | NA | NA | 8 |
|  |  |  |  |  |  | **Total** | **87** |
|  |  |  |  |  |  |  |  |
| New Zealand | Pukekohe | 2006 | Adults | NA | NA | NA | 12 |
|  |  |  |  |  |  |  |  |
| Brazil | Matto Grosso | 2012 |  | NA | NA | Tomato, Cotton, Perl millet, Maize, Sunflower | 24 |
|  | Maranhao | 2012 |  | NA | NA | Cotton | 3 |
|  | Parana | 2012 |  | NA | NA | Wheat | 3 |
|  | Minas Gerais | 2012 |  | NA | NA | Cotton | 7 |
|  | Goais | 2012 |  | NA | NA | Tomato, Pearl millet, Bean, Cotton | 55 |
|  | Bahia | 2012 |  | NA | NA | Cotton | 2 |
|  |  |  |  |  |  | **Total** | **94** |
|  |  |  |  |  |  |  |  |
| Helicoverpa zea |  |  |  |  |  |  |  |
|  |  |  |  |  |  |  |  |
| Brazil | Matto Grosso | 2012 |  |  |  | Cotton | 13 |
|  | Goais* |  |  |  |  | Cotton/Tomato | 14 |
|  |  |  |  |  |  |  |  |
|  | Matto Grosso | 2006 |  |  |  | Cotton | 16 |
|  |  |  |  |  |  |  |  |
| USA | North Carolina | 2002 |  |  |  | Cotton | 16 |
|  |  |  |  |  |  |  |  |
| * Potential hybrid identified from this location | | | | | | | |
