# Supplementary material for: Multiple recombination events between two cytochrome P450 loci contribute to global pyrethroid resistance in Helicoverpa armigera
Source: PLoS One. 2018 Nov 1;13(11):e0197760. doi: 10.1371/journal.pone.0197760 (PMC6211633; doi:10.1371/journal.pone.0197760)
Supplement: S2 Table — (DOCX) [file pone.0197760.s008.docx]

**S2 Table. Primers used in this work.**

| Primer name | 5' to 3' |  |
| --- | --- | --- |
| CYP337B1-F | AATAATAAGCAACGCCAATGACTTAC | Diagnostic B1 forward |
| CYP337B1-R | TGCAATAACAACTATTACATTTTGAATAAA | Diagnostic B1 reverse |
| CYP337B2-F | AATAATAACCAGTCCAAACGATGTGT | Diagnostic B2 forward |
| CYP337B2-R | CGCAATTATAGTCACTATGATTGCATATA | Diagnostic B2 reverse |
| CYP337B3-F | AATAATAACCAGTCCAAACGATGTGT | Diagnostic B3 forward |
| CYP337B3-R | TGCAATAACAACTATTACATTTTGAATAAA | Diagnostic B3 reverse |
| CYP337B3_intR | GGTATGCAATTGTCCTGT | Intron B3 reverse |
| CYP337B1_FL_F | CACAATGGTATTGTCAATATTAT | Full length B1 forward |
| CYP337B2_FL_F | CACAATGGTGTTCATAATAT | Full length B2 forward |
| CYP337B3_FL_F | CACAATGGTGTTCGTGATAT | Full length B3 forward |
| CYP337B1_FL_R | TATATCTCTTAACTTTAATTCATAAC | Full length B1 reverse |
| CYP337B2_FL_R | TATATCTCTTAACTTAAATTCATAATG | Full length B2 reverse |
| CYP337B3_FL_R | TATGTCTCTTAACTTTAATTCATAACA | Full length B3 reverse |
| CYP337B1_cloning F | CTTTATGTACTAGGGTGAAGT | B1 cloning forward |
| CYP337B1 cloning R | AAAAAGTGTACGCGACATTCA | B1 cloning reverse |
| CYP337B2 cloning F | CTGGAGCATCATTACATCACCGT | B2 cloning forward |
| CYP337B2 cloning R | CATCTTGCGTACTTTATCATCAGAACA | B2 cloning reverse |
| CYP337B3 cloning (GR/BF) F | CGTGCACATCGCGCTCA | B3 cloning forward |
| CYP337B3 cloning (GR/BF) R | GTTACGTAGCAGTTGTACGCAA | B3 cloning reverse |
| CYP337B3 cloning (KOR) F | CTGGAGCATCATTACATCACCGT | B3 cloning forward |
| CYP337B3 cloning (KOR) R | AAAAAGTGTACGCGACATTCA | B3 cloning reverse |

BF, Burkina Faso; GR, Greece; KOR, Korea
