# Supplementary material for: Multiple recombination events between two cytochrome P450 loci contribute to global pyrethroid resistance in Helicoverpa armigera
Source: PLoS One. 2018 Nov 1;13(11):e0197760. doi: 10.1371/journal.pone.0197760 (PMC6211633; doi:10.1371/journal.pone.0197760)
Supplement: S3 Table — (DOCX) [file pone.0197760.s009.docx]

**S3 Table. Specific sequence identifiers, gene names, origin and NCBI numbers used for the maximum likelihood estimate of phylogenetic relationships.**

| Sequence Ident. | Gene name | Origin | NCBI number |
| --- | --- | --- | --- |
| B1-BF1 Burkina faso | *CYP337B1-BF1* | Burkina faso | KX958380 |
| B1-BF2 Burkina faso | *CYP337B1-BF2* | Burkina faso | KX958381 |
| B1-HC Korea | *CYP337B1-HC* | Hongcheon, Korea | KX958385 |
| B1-PC Korea | *CYP337B1-PC* | Pyeongchang, Korea | KX958386 |
| B1-TWBv1 Australia | *CYP337B1-v1* | Toowoomba, Australia | JQ284023, JQ995291 (BAC 18J13) |
| B1-AN02v1like Australia | *CYP337B1-v3* | Australia | JQ284024 |
| B1-AN02v4like Australia | *CYP337B1-v4* | Australia | JQ284025 |
| B1-GR1 Greece | *CYP337B1-GR1* | Greece | KX958382 |
| B1-GR2 Greece | *CYP337B1-GR2* | Greece | KX958383 |
| B1-GR3 Greece | *CYP337B1-GR3* | Greece | KX958384 |
| B3-v1 Australia | *CYP337B3-v1* | Toowoomba, Australia | JQ995292.1 |
| B3-v2 Australia | *CYP337B3-v2* | Faisalabad, Pakistan | KJ636466.1 |
| B3-v3 China | *CYP337B3-v3* | Qianjiang ,China | KM675665.1 |
| B3-v4 China | *CYP337B3-v4* | Luoyang, China | KM675666.1 |
| B3-v5 Uganda | *CYP337B3-v5* | Uganda | KX958388 |
| B3-v6 Uganda | *CYP337B3-v6* | Uganda | KX958389 |
| B3-v7 China | *CYP337B3-v7* | China | KX958390 |
| B3-v8 China | *CYP337B3-v8* | China | KX958391 |
