# Supplementary material for: Multiple recombination events between two cytochrome P450 loci contribute to global pyrethroid resistance in Helicoverpa armigera
Source: PLoS One. 2018 Nov 1;13(11):e0197760. doi: 10.1371/journal.pone.0197760 (PMC6211633; doi:10.1371/journal.pone.0197760)
Supplement: S4 Table — (DOCX) [file pone.0197760.s010.docx]

**S4 Table. Sequencing and alignment statistics for the 12 individuals used to demonstrate the selective sweep around *CYP337B3v1*.**

| **Sample** | **Total reads** | **Reads aligned** | **Ambiguous alignments (%)** | **Low quality reads discarded (%)** |
| --- | --- | --- | --- | --- |
| HM0001 | 81,530,580 | 2,230,469 | 13.04 | 0.06 |
| HM0002 | 18,981,842 | 494,528 | 13.72 | 0.27 |
| HM0003 | 21,993,262 | 589,945 | 13.98 | 0.23 |
| HM0004 | 15,604,520 | 369,719 | 14.07 | 0.29 |
| M0086 | 50,516,412 | 1,285,051 | 13.29 | 0.27 |
| M0163 | 58,164,108 | 1,444,699 | 13.57 | 0.08 |
| M0243 | 46,216,454 | 1,193,149 | 14.16 | 0.08 |
| M0260 | 41,203,824 | 1,066,945 | 13.70 | 0.28 |
| M0261 | 42,389,756 | 1,051,542 | 13.82 | 0.07 |
| M0273 | 64,402,336 | 1,518,666 | 14.16 | 0.08 |
| M0276 | 14,847,962 | 380,380 | 13.75 | 0.07 |
| M0299 | 20,651,186 | 543,509 | 13.78 | 0.08 |
