# Supplementary material for: Multiple recombination events between two cytochrome P450 loci contribute to global pyrethroid resistance in Helicoverpa armigera
Source: PLoS One. 2018 Nov 1;13(11):e0197760. doi: 10.1371/journal.pone.0197760 (PMC6211633; doi:10.1371/journal.pone.0197760)
Supplement: S1 File — (DOCX) [file pone.0197760.s011.docx]

**S1 File. Intron sequence data used for the maximum likelihood estimate of phylogenetic relationships.**

>CYP337B3-QJ_v7 of Helicoverpa armigera from Qianjiang (strains: QJ) (clone QJ11-1)

GTACCATGGAATCACCATTCGTTGAATTAGCATTTAGAGCTTTACGACCATCGTTTATTCAAAATGTAATAGTTGTTATTGCAAATACTTTCCCTAGATTGTACAAACTCTTGCAGTTGAAACTTTTTGGTGAACATGAAGAATTTTTTGTGGGTGCAGTGAAAACTGTTTTAGAAAGTAGGAGGCATGATACTACTAAAAGATATGATTTTATTGAGATTTGCTTGGAACTACAGAAGAATGGAATAATGCAGGACTTCTCAACTGGATATAAGTTGGAGCCGACCGACGAACTGATGGCTGCTCAAGCATTCTTCTTTTTTGTGGCTGGTGCAGACACCTCTGCTAACACAATGCATTATTCTTTGTTAGAGCTGTCAAACAATCCAAAAATCTTGGCAAAACTACATGAGGAAATTGACAAAGTTTTCGAAGGCGGAGCTGGGGAACTAACTTATAATGACATTGATAAGTTACAATATTTAGATCAGGTTATAAATGAAGCGATGAGAAAGTATCCTCCAGTAGGTGTCATGCAAAGATTGTGCACTAAAGATACAGTTTTACCTTCTGGTATACCCATAGCAAAAGGTAACACAATAATGATTCCAGTGTTCGGTCTTCACAGAGACGAAAAATACTTCGATGACCCACATGTATTTGATCCGGATAGATTTTCACCAGAAAATGTATCCAAAATCAAGAATTATGCTTATTTACCTTTTGGTGAAGGAAACCGCATTTGTATTGGTGAGTATTAAACATTTATACATATGTATTGTTAATATTGTAAATATATTTGTGTAACTCATACATTTAAGATTCTAGTGAAGGCATAAATCTTATTTAGGTACCTAGTACGCAACGGGCGTGCTAGTCTTTTTATGGTTCAAAACAATTGTTCAATGATCGACCATAATCATCAATACTTATTCTACCCCATTCATTCAATCATTGTCTCATAGTAAGTAATCATAAAAATACATAAATGAATGGTTATCATTCCCTTTGTTTAGTTTTACTAATTGCATCATCCGATAAATGAGTGAGTGATCTGATAATGCATTATTTAATTTAAATGATTATGACGGCCTAATTCGGTTATCATGAATCATTTTAATCATTAGTTAGCAAATATTTTTCTTTAATCTTGGCTTATCTTGACAAACTTTATACCTAGTACTAATTGTCTGTGTTTTTACCTTTTACTATTTATCCCTATTTGTTATCTCAAGTGTATAATTATAGTACTTACGACAATAGTTTACCACAAAGTTATATAATGTAGTATGTAAATAGAAGTTGATAAAACAACGACTAGTCTCTACCTGAGTGCAGTGGCACGCAGTGGTTTGTACACAATATTAATATTCTGGTTTCATCTGACTTATTGAATTACGTCCCTAATACATACAAGGTCTTTATCTAACACTGTAGGTTTATTTTATAACTAGGTGCTTCCAGCGGTTTAACTCTCAACCTACACATAAAAGTAGCATCGATAATGAGCTGCCTACTTACCACTATAAGAATTTCTCAAATTACCTCAGCTATCCCAGGTTCAGATATAATGGTTCAGGCATAGATGATGATGATAACTCAGATATTTTTTATATTCTAAAGTACTTTTCCATCTTGTTCCAGGTGTACGCTTCGCTCGCCTCCAAGTGAAAGCGGGTCTCGCCTGGCTGTTACGTCGTTTTACCTTAGTCGAACAGGACAATTGCATACCAGAATTTGAGAAAAGTCCATTCGGTCTACGAAGTCCCGATGCTCGTTATGAATTAAAGTTAAGAGACATATAA

>CYP337B3-LY-A_v8 of Helicoverpa armigera from Luoyang and Xuzhou (strains: LY, XZ) (clone LY1-1a)

GTACCATGGAATCACCATTCGTTGAATTGGCATTTAGAGCTTTACGACCATCGTTTATTCAAAATGTAATAGTTGTTATTGCAAATACTTTCCCTAGATTGTACAAACTCTTGCAGTTGAAACTTTTTGGTGAACATGAAGAATTTTTTGTGGGTGCAGTGAAAACTGTTTTAGAAAGTAGGAGGCATGATACTACTAAAAGATATGATTTTATTGAGATTTGCTTGGAACTACAGAAGAATGGAATAATGCAGGACTTCTCAACTGGATATAAATTGGAGCCGACTGACGAACTGATGGCTGCTCAAGCATTCTTCTTTTTTGTGGCTGGTGCAGACACCTCTGCTAACACAATGCATTATTCTTTGTTAGAGCTGTCAAACAATCCAAAAATCTTGGCAAAACTACATGAGGAAATTGACAAAGTTTTCGAAGGCGGAGCTGGGGAACTAACTTATAATGACATTGATAAGTTACAATATTTAGATCAGGTTATAAATGAAGCAATGAGAAAGTATCCTCCAGTAGGTGTCATGCAAAGATTGTGTACTAAAGATACAGTTTTACCTTCTGGTATACCCATAGCAAAGGGTAACACAATAATGATTCCAGTGTTCGGTCTTCACAGAGACGAAAAATACTTTGATGACCCACACGTATTTGATCCGGATAGATTTGCACCAGAAAACGTATCCAAAATCAAGAATTATGCTTATTTACCTTTTGGTGAAGGAAACCGCATTTGTATTGGTGAGTATTCAACATTTACTTATAACTTAATTTAAATATATTTTTGTAACTTTATATTTAAGGTTCTAGAGAAGGCATAAATCTTATTTAGCTTTGATCAGTTATTCTAAAGTAGGTACCTAGTACGCAATGGGCGTGCTAGTCTTTTTATGGCCCAAAACAATTGTTCAATGAACGGTCATAATCTTCCATACTTATTCCACCCCATTCATTCAATCATTGTCTCATATTAAGTAGTCATAAAAATACATAAATGAATAGTTATCATTCCCTTTGTTTAGTTTTACTAATTGCATCATCCGATAAATCAGTGAGTGATCTGATAATGCATTATTTAATTTAAATGATAATGACGGCCTAATTCGGTTATCATTAGTTATTTAATCGTTAGTTAGCAAATATTTTTCTTTAATCTTGGCTTATCTTGACAAACTTTATACCTAGTACTAATTGTTTGTCTGTTTATTTTTACCTATTATTATGTATCCCTATCTCAGGTGCGTTGTTTTAAAAATAGTATTTACGACAATAGTTTACCACAAAGTTATATAATGCACTATGTAAATAGTCTGAAGTTGATAAAACAACGACTAGTCTACATGAGTGCAGTGGTTTGTACACAATATCTATTAATATTCTGGTTTCATCTGACTTATTGAATTACGTCGCTATAACATACAAGGTTTTTATCTAATACTGTACGGTTATATTATAACTAGCTTCCGCCAGTGGTTTAACTCTCAACCCACACATAAAAAGTAGCATCGATTATAATGTTGCTGCTTAACACTTTAAGAATTTCTCAAATTACCCCAGCTATCCCAGGTTCAGATATAATGGTTCAGGTATAGATGATGATGATAACTGAGATACTTTTTTTTATGTTTTAAAGTACTTTTCAATCTTGTTCCAGGTGTACGCTTCGCTCGCCTCCAAGTGAAAGCGGGTCTCGCCTGGTTGTTACGTCGTTTTACTCTAGTCGAACAGGACAATTGCATACCAGAATTCGAGAAAAGTCCATTCGGTCTACGAAGTCCCGATGCTCGTTATGAATTAAAGTTAAGAGACATATAA

>CYP337B3v2 gi|657202136|gb|KJ636466.1| Helicoverpa armigera strain Faisalabad cytochrome P450 337B3v2 (CYP337B3) gene, CYP337B3-2 allele, complete cds

GTACCATGGAATCACCATTCGTTGAATTGGCATTTAGAGCTTTACGACCATCGTTTATTCAA

AATGTAATAGTTGTTATTGCAAATACTTTCCCTAGATTGTACAAACTCTTGCAGTTGAAACTTTTTGGTG

AACATGAAGAATTTTTTGTGGGTGCAGTGAAAACTGTTTTAGAAAGTAGGAGGCATGATACTACTAAAAG

ATATGATTTTATTGAGATTTGCTTGGAACTACAGAAGAATGGAATAATGCAGGACTTTTCAACTGGATAT

AAATTGGAGCCGACCGACGAACTGATGGCTGCTCAAGCATTCTTCTTTTTTGTGGCTGGTGCAGACACCT

CTGCTAACACAATGCATTATTCTTTGTTAGAGCTGTCAAACAATCCAAAAATCTTGGCAAAACTACATGA

GGAAATTGACAAAGTTTTCGAAGGCGGAGCTGGGGAACTAACTTATAATGACATTGATAAGTTACAATAT

TTAGATCAGGTTATAAATGAAGCAATGAGAAAGTATCCTCCAGTAGGTGTCATGCAAAGATTGTGTACTA

AAGATACAGTTTTACCTTCTGGTATACCCATAGCAAAGGGTAACACAATAATGATACCAGTGTTCGGTCT

TCACAGAGACGAAAAATACTTTGATGACCCACACGTATTTGATCCGGATAGATTTTCACCAGAAAACGTA

TCCAAAATCAAGAATTATGCTTATTTACCTTTTGGTGAAGGAAACCGCATTTGTATTGGTGAGTACCTAT

TAAACATCTATACATATGTATTGTTAATCTTCATATATATAAAAATGAATTGCTGTTCGTTAGTCTTACT

AAAACTCGAGTATGGCTGGACCGATTTGGCTTATTTTGGTGTTAAAATGTTTGTAGAGGTCCAGGGAAGG

TATAAACGATACGAAGTTTGCGGGATCAGCTAGTATTGTAAATATATTTGTGTAACTTATACTTGTTTAG

CTTTGATCAGTTATTCTAAAGAAGGTACCTATTACGCAATGTGCGTCCTAGTCTTTTTATGGATCAAAAC

AATTGTTCAATGATCGACCATAATCTTCAATACTTATTCTACCCCATTCATTCAATCATTGTGTCATATT

AAGAAGTCATAAAAATACATATATGAATAGTTATCATTCCCTTTGTTTAGTTTTACTAATTGCATCATCC

GATAAATGAGTGAGTGATCTGATAATGCATTATTTAATTTTTTAATTTGAATGATAATGACGGCCTAATT

CGGTTATCATAAATCATTAAATCATTAGTTAGCAAATATTTTTCTTTAATCTTTGCTAATCTTGACAAAC

TTTATACCTAGTACTAATTATTAGTTATTTTGACTATTCCTACTTATCCCTATTTATTATCTCAAGTGCG

TATTTGTTTAATAGTAGTATTTACGACAATAGTTTACCACAAAGTTATATAAGTACTATGTAAATAGTCT

GCAGTTGATAAAACAACTACTAGTCTCTACCTGAGTGCAGTGGTTTGACAATACCGATTAATATTCTGGT

TTCATCTGACTTATTGAATTACGTCCCTATTACATACAAGGTCTCTATCTAACACTGTAGGTTTATTTTA

TAACTAGCTTGCGCCAGCGGTTTAATTCTCAACCTACACATAAAAGTAGCATCAATAATAATACAGCTAC

TTAACACTATAAGAATTTCTCAAATCACCCCAGTTATCCCAGGTTAAAACTGAGATATTTTTTATGTTCT

AAAGTACTTTTCCGTCTTGTTCCAGGTGTACGCTTCGCTCGCCTCCAAGTGAAAGCGGGTCTCGCCTGGC

TGTTACGTCGTTTTACCTTAGTCGAACAGGACAATTGCATACCAGAATTCGAGAAAAGTCCATTTGGCCT

CCGAAGTCCCGATGCTCGTTATGAATTAAAGTTAAGAGACATATAA

>CYP337B3v1 gb|JQ995292.1|:79754-82369 Helicoverpa armigera clone BAC 33J17 cytochrome P450 337B3v1 gene, complete cds

GTACCATGGAATCACCATTCGTTGAATTGGCATTTAGAGCTTTACGACCATCGTTTATTCAAAATG

TAATAGTTGTTATTGCAAATACTTTCCCTAGATTGTACAAACTCTTGCAGTTGAAACTTTTTGGTGAACA

TGAAGAATTTTTTGTGGGTGCAGTGAAAACTGTTTTAGAAAGTAGGAGGCATGATACTACTAAAAGATAT

GATTTTATTGAGATTTGCTTGGAACTACAGAAGAATGGAATAATGCAGGACTTCTCAACTGGATATAAAT

TGGAGCCGACCGACGAACTGATGGCTGCTCAAGCATTCTTCTTTTTTGTCGCTGGTGCAGACACCTCTGC

TAACACAATGCATTATTCTTTGTTAGAGCTGTCAAACAATCCAAAAATCTTGGCAAAACTACATGAGGAA

ATTGACAAAGTTTTCGAAGGCGGAGCTGGGGAACTAACTTATAATGACATTGATAAGTTACAATATTTAG

ATCAGGTTATAAATGAAGCAATGAGAAAGTATCCTCCCATAGGTGTCATGCAAAGATTGTGTACTAAAGA

TACAGTTTTACCGTCTGGTATACCCATAGCAAAGGGTAACACAATAATGATTCCAGTGTTCGGTCTTCAC

AGAGATGAAAAATACTTCGATGACCCACATGTATTTGATCCGGATAGATTTTTACCAGAAAACGTATCCA

AAATCAAGAATTACGCTTATTTACCTTTTGGTGAAGGAAACCGCATTTGTATTGGTGAGTATTAAACATT

TATACATATGTATTGTTAATATTGTAAATATATATGTGTAACTCATACATTTAAGATTGTAGTGAAGGCA

TAAATCTTATTTAGCTTTGATCAGTTATTCTGAAGTAGGTACCTAGTACGCAATGGGCGTGCTAGTCTTT

TTATGGCCCAAAACAATTGTTCAATGAACGGTCATAATCTTCCATACTTATTCCACCCCATTCATTCAAT

CATTGTCTCATAGTAAGTAGTCATAAAAATACATAAATGAATAGTTATCATTCCTTTTGTTTAGTTTTAC

TAATTGCATCATCCGATAAATCAGTGAGTGATCTGATAATGCATTATTTAATTTAAATGATAATGACGGC

CTAATCCGGTTATCATTAATTATTTAATCATTAGTTAGCAAATATTTTTCTTTAATCTTGGCTTATCTCG

ACAAACTGTATACCTAGTACTAATTATTCGTCTATTTATTTTTTCCTATTATTATGTATCCCTATCTCAG

GTGCGTCGTTTTAATTATAGTACCTACGACAATAGTTTACAACAAAGGTATAAGTTACTATGGAAATAGT

CTGAAGTTGATAAAACAACGACTAGTCTCTACCTGCAGTAGTTTGTACACAATATTAATATTCTGGTTTC

ATCCGACTTATTGAATTACGTCTCTATTACATACAATGTCTTTATCTAACACTGTAGGGTTATTTTATAA

CTAGGTGCCACCTGCGGTTTAATTCTCAACTCATATTGCGCTCACATAAAAATTAGCATCGATAATAATG

TGGCTACTTAACACTACAAGAATTTCTCAAATTACCCCAGCTATCCCAGGTTCAGATATAATGGTTCAGG

CATAGACGATAATGATAACTGAGATACTTTTTTATGTTCTAAAGTACTTTTCCATCTTGTTCCAGGTGTA

CGCTTCGCTCGCCTCCAAGTGAAAGCGGGTCTCGCCTGGCTGTTACGTCGTTTTACCTTAGTCGAACAGG

ACAATTGCATACCAGAATTCGAGAAAAGTCCATTTGGCCTCCGAAGTCCCGATGCTTGTTATGAATTAAA

GTTAAGAGACATATAA

>CYP337B1-HC of Helicoverpa armigera from Hongcheon, Korea (strain: HC)

GTACCATGGAATCACCATTCGTTGAATTGGCATTTAGAGCTTTGCGACCATCGTTTATTCAAAATGTAATAGTTGTTATTGCAAATACTTTCCCTAGATTGTACAAACTCTTGCAGTTGAAACTTTTTGGTGAACATGAAGAATTTTTTGTGGGGGCAGTGAAAACTGTTTTAGAAAGTAGGAGGCATGATACTACTAAAAGATATGATTTTATTGAGATTTGCTTGGAACTACAGAAGAATGGAATAATGCAGGACTTCTCAACTGGATATAAATTGGAGCCGACCGACGAACTGATGGCTGCTCAAGCATTCTTCTTTTTTGTCGCTGGTGCAGACACCTCTGCTAACACAATGCATTATTCTTTGTTAGAGCTGTCAAACAATCCAAAAATCTTGGCAAAACTACATGAGGAAATTGACAAAGTTTTCGAAGGCGGAGCTGGGGAACTAACTTATAATGACATTGATAAGTTACAATATTTAGATCAGGTTATAAATGAAGCAATGAGAAAGTATCCTCCAGTAGGTGTCATGCAAAGATTGTGTACTAAAGATACAGTTTTACCTTCTGGTATACCCATAGCAAAGGGTAACACAATAATGATACCAGTGTTCGGTCTTCACAGAGACGAAAAATACTTTGATGACCCACACGTATTTGATCCGGATAGATTTTCACCAGAAAACGTATCCAAAATCAAGAATTATGCTTATTTACCTTTTGGTGAAGGAAACCGCATTTGTATTGGTGAGTATTAAACATTTATACATACGTATTGTTAATATTGTAAATATATTTGTGTAACTTATACACATTTAATATTGTAAATATATAAATGTATGAGTTATACATAACTCATACATTTAAGATTCTAGAGAAGGCATAAATCTTATTAAGCTTTGATTAGTTTTTCAAAAGTAGGTACCTAGTACGCAATGGGCGTGCTAGTCTTTTTATGGCCCAAAACAATTGTTCAATGATCGACCATAATCATCAATACTTATTCTACCCCATTCATTCAATCATTGTCTCATAGTAAGTAGTCATAAAAATACATAAATGAATAGTTATCATTCCCTTTGTTTAGTTTTACTAATTGCATCATCCGATAAATGAGTGAGTGATCTGATAATGCATTATTTAATTTAAATGATAATGACGGCCTAATTCGGTTATCATTAATCATTTTAATAATTAGTTAGCAAATATTTTTCCTTAATCTTGGCTTATCTTGACAAACTTTATACCTAGTACTAATTGTTTGTCTATTTCTTTATACCTATTACTGTTGCTCGAAGCCTATTACAAAGTATCCCTATTTATAATCTCAAGTGCGTCGTTTAAATTAATTGTAGTTGTTATGACACTACTCTAGCCCAAAGATGTACGTCTATAGGTATATATAAAAGAAAGTCGTGTTAGTTACTCCACGGATAACTCAAGAACGCCTGAACCGATTTAGCTGAAAAGTTTTTGTCACCATCCGGCTACGGAAAGCAGTTATCTCGGGACGCGGGTGAAACCGCGGGCGGAAAGCTAGTACTATATAAATAGTCTTAAGAGAACAACTACTAGTCTCTACATGAGTGCAGTGGTTTGTACACAATAATAATATTCTGGTTTCATCTGACTTATTGAATTACGTCCCTATTACATACAAGGTCTCTATGATCTAACGCTGTAGGGTTATTTTATAACTAGCTTCCGCCAGCGGTTTAATTCTCAACTCATATTGCGCTCACATAAAAATTAGCATCGATAATAATGTGGCTACTTAACACTATAAGAATTTCTCAAATTACCCCAGCTATCCCAGGTTCAGATATAATGGTTCAGGTATAGATGATGATGATAACTGAGATACTTTTTTATGTTCTAAAGTACTTTTCTATCTTGTTCCAGGTGTACGCTTCGCTCGCCTCCAAGTGAAAGCGGGTCTCGCCTGGCTGTTACGTCGGTTTACTTTAGTCGAACAGGACAATTGCATACCAGAATTCGAGAAAAGTCCATTTGGCCTCCGAAGTCCCGATGCTCGTTATGAATTAAAGTTAAGAGACATATAA

>CYP337B1-PC of Helicoverpa armigera from Pyeongchang, Korea (strain: PC)

GTACCATGGAATCACCATTCGTTGAATTGGCATTTAGAGCTTTGCGACCATCGTTTATTCAAAATGTAATAGTTGTTATTGCAAATACTTTCCCTAGATTGTACAAACTCTTGCAGTTGAAACTTTTTGGTGAACATGAAGAATTTTTTGTGGGTGCAGTGAAAACTGTTTTAGAAAGTAGGAGACATGATACTACTAAAAGATATGATTTTATTGAGATTTGCTTGGAACTACAGAAGAATGGAATAATGCAGGACTTCTCAACTGGATATAAATTGGAGCCGACCGACGAACTGATGGCTGCTCAAGCATTCTTCTTTTTTGTGGCTGGTGCAGACACCTCTGCTAACACAATGCATTATTCTTTGTTAGAGCTGTCAAACAATCCAAAAATCTTGGCAAAACTACATGAGGAAATTGACAAAGTTTTCGAAGGCGGAGCTGGGGAACTAACTTATAATGACATTGATAAGTTACAATATTTAGATCAGGTTATAAATGAAGCAATGAGAAAGTATCCTCCAGTAGGTGTCATGCAAAGATTGTGTACTAAAGATACAGTTTTACCTTCTGGTATACCCATAGCAAAGGGTAACACAATAATGATTCCAGTGTTCGGTCTTCACAGAGACGAAAAATACTTTGATGACCCACACGTATTTGATCCGGATAGATTTGCACCAGAAAACGTATCCAAAATCAAGAATTATGCTTATTTACCTTTTGGTGAAGGAAACCGCATTTGTATTGGTGAGTACCTATTAAACATTTATACATTATGTATTGTTAATCTTCATATATATAAAAATGAATTGCTGTTCGTTAGTCTTACTAAAACTCGAGTATGGCTGGGCCGATTTGGCTTATTTTGGTGTTAAAATGTTTGTAGAGGTCCAGGGAAGGTTTAAACGATACGAAGTTTGCGGGATCAGCTAGTATTGTAAATATATTTGTGTAACTTATACTTATTTAGCTTTGATCAGTTATTCTAAAGTAGGTACCTAGTACGCAATGGGCGTGCTAGTCTTTTAATGGCCCAAAACAATTGTTCAATGAACGGTCATAATCTTCCATACTTATTCCACCCCATTCGTTCAATCAGTGTCTCATAAAGTAGTCATAAAAATACATAAATGAATGGTTATCATTCCCTTTGTTTAGTTTTACTAATTGCATCATCCGATAAATGAGTGAGTGATCTGATAATGCATTATTTAATTTAAATGATTATGACGGCCTAATTCGGTTATCATTAATTATTTAATCGTTAGTTAGCAAATATTTTTCATTAATCTTGGCTTATCTTGACAAAATACCTAGTACTAATTGTATGTCTATTTCTTTTTACCTATTACTGTTGCTCGAAGCCTATTACAAAGTATCCCTATTTATAATCTCAAGTGCGTCGTTTAAATTAATTGTAGTTGTTATGACACTGCTCTAGCCCAAAGATGTACGTCTATAGGTATATATAAAAGAAAGTCGTGTTAGTTACTCCACTTATAACTCAAGAACGCCTGAACCGATTTAGCTGAAAAGTTTTGTCACCATCCGGCTACGGGAAGCAATTATCTCGGACGTGGGTGAAACTGCGTGCGGAAAGCTAGTACTATATAAATAGTCTTAAGAGAACAACGACTAGTCTCTACCTGAGTGCAGTGGTTTGTACACAATATTAATTATTCTGGTTTCATCTGACTTATTGAATTACGTCCCTATTACATACAAGGTCTTTATCTAACACTGTAGTGTTATTTTATAACTAGCTTCCGCCAGCGGTTTAACTCTCAACCCACTCAATAAAAAATAGCATCGATAATAGTACTTAACACTATAAGAATTTCTCAAATTACCCCAGATATCCCAGATTCAGATATAATGGTTCAGGCATAGATGATGATAACTGAGATACTTTTTTATGTTTTAAAGTACTTTTCAATCTTGTTCCAGGTGTACGCTTCGCTCGCCTCCAAGTGAAAGCGGGTCTCGCCTGGCTGTTACGTCGGTTTACCTTAGTCGAACAGGACAATTGCATACCAGAATTCGAGAAAAGTCCATTTGGCCTCCGAAGTCCCGATGCTCGTTATGAATTAAAGTTAAGAGACATATAA

>CYP337B1-BF2 of Helicoverpa armigera from Burkina Faso (strain: BF) GTACCATGGAATCACCATTCGTTGAATTGGCATTTAGAGCTTTGCGACCATCGTTTATTCAAAATGTAATAGTTGTTATTGCAAATACTTTCCCTAGATTGTACAAACTCTTGCAGTTGAAACTTTTTGGTGAACATGAAGAATTTTTTGTGGGTGCAGTGAAAACTGTTTTAGAAAGTAGGAGGCATGATACTACTAAAAGATATGATTTTATTGAGATTTGCTTGGAACTACAGAAGAATGGAATAATGCAGGACTTCTCAACTGGATATAAATTGGAGCCGACCGACGAACTGATGGCTGCTCAAGCATTCTTCTTTTTTGTCGCTGGTGCAGACACCTCTGCTAACACAATGCATTATTCTTTGTTAGAGCTGTCAAACAATTCAAAAATCTTGGCAAAACTACATGAGGAAATTGACAAAGTTTTCGAAGGGGGAGCTGGCGAACTAACTTATAATGACATTGATAAGTTACAATATTTAGATCAGGTTATAAATGAAGCAATGAGAAAGTATCCTCCAGTAGGCGTCATGCAAAGATTGTGTACTAAAGATACAGTTTTACCTTCTGGTATACCAATAGCAAAAGGCAACACAATAATGATTCCAGTGTTCGGTCTTCACAGAGACGAAAAATACTTCGATGACCCACATGTTTTTGATCCGGATAGATTTTTACCAGAAAACGTATCCAAAATCAAGAATTATGCTTATTTACCTTTTGGTGAAGGAAACCGCATTTGTATTGGTGAGTATTAAACATTTACTTATTCATATTGTAAATATATTTGTGTAACTTATACATTTAAGGTTCTAGAGAAGGCATAAATCTTATTTAGCTTTGATCAGTTATTCTAAAGTAGGTACCTAGTACGCAACGGGCGTGCTAGTTTTTTATGGCCCAAAACAATTGTTCAATGATCGACCATAATCTTCAATACTTATTCTACCCCATTCATTCAATCATTGTCTCATAGTTAGTAGTCATAAAAATACATAAATGAATAGTTATCATTCCCTTTGTTTAGTTTTACTAATTGCATCATCCGATAAATGAGTGAGTGATCTGATAATGCATTATTTAATTTTTAATTTAAATGATAATGACGGCCTAATTCGGTTATCATTAATCATTAAATCATTAGTTAGCAAATATTTTTCTTTAATCTTTGCTAATCTTGACAAACTTTATACCTAGTACTAATTATTTATTTATTCTTACCTATTACTACTTATCCCTATTGATTATCTCAAGTGCGTATTTGTTTAATAGTAGTATTTACGACAATAGTTTACCACAAAGTTATAGGTATTATGCACTATGTAAATAGTCTGAAGTTGATAAAACAACGACTAGTCTCTACCTGAGTGCAGTGGTTTGTACACAATATTATTATTCTGGTTTCATCTGACTTATTGAATTACGTCGCTATTACATACAAGGTCTTTATCTAACACTGTAGTGTTATTTTATAACTAGCTTCCGCCAGCGGTTTAACTCTCAACTTACACATAAAAGTAGCATCAATAATAATGTGGCTATTTAACACTGAAAAAAAATCTCAAATCACCCCAGTTATCCCAGGTTCAGATATAATGGTTCAGGTATAGATGATGATGATACCTTAGGTATTTTTTATGTTCTAAAGTACTTTTACATCTTGTTCCAGGTGTACGCTTCGCTCGCCTCCAAGTGAAAGCGGGTCTCGCCTGGCTGTTGCGTCGTTTTACCTTAGTCGAACAAAACAATTGCCTACCAGAATTCGAGAAAAGTCCATTCGGTCTACGAAGTCCCGATGCTCGTTATGAATTAAAGTTAAGAGACATATAA

>CYP337B1TWBv1

GTACCATGGAATCACCATTCGTTGAATTGGCATTTAGAGCTTTACGACCATCGTTTATTCAAAATGTAATAGTTGTTATTGCAAATACTTTCCCTAGATTGTACAAACTCTTGCAGTTGAAACTTTTTGGTGAACATGAAGAATTTTTTGTGGGTGCAGTGAAAACTGTTTTAGAAAGTAGGAGGCATGATACTACTAAAAGATATGATTTTATTGAGATTTGCTTGGAACTACAGAAGAATGGAATAATGCAGGACTTTTCAACTGGATATAAATTGGAGCCGACCGACGAACTGATGGCTGCTCAAGCATTCTTCTTTTTTGTGGCTGGTGCAGACACCTCTGCTAACACAATGCATTATTCTTTGTTAGAGCTGTCAAACAATCCAAAAATCTTGGCAAAACTACATGAGGAAATTGACAAAGTTTTCGAAGGCGGAGCTGGGGAACTAACTTATAATGACATTGATAAGTTACAATATTTAGATCAGGTTATAAATGAAGCAATAAGAAAGTATCCTCCCATAGGTGTCATGCAAAGATTGTGTACTAAAGATACAGTTTTACCGTCTGGTATACCCATAGCAAAGGGTAACACAATAATGATTCCAGTGTTCGGTCTTCACAGAGATGAAAAATACTTCGATGACCCACATGTATTTGATCCGGATAGATTTTTACCAGAAAACGTATCCAAAATCAAGAATTACGCTTATTTACCTTTTGGTGAAGGAAACCGCATTTGTATTGGTGAGTATTAAACATTTATACATATGTATTGTTAATATTGTAAATATATATGTGTAACTCATACATTTAAGATTGTAGTGAAGGCATAAATCTTATTTAGCTTTGATCAGTTATTCTGAAGTAGGTACCTAGTACGCAATGGGCGTGCTAGTCTTTTTATGGCCCAAAACAATTGTTCAATGAACGGTCATAATCTTCCATACTTATTCCACCCCATTCATTCAATCATTGTCTCATAGTAAGTAGTCATAAAAATACATAAATGAATAGTTATCATTCCCTTTGTTTAGTTTTACTAATTGCATCATCCGATAAATCAGTGAGTGATCTGATAATGCATTATTTAATTTAAATGATAATGACGGCCTAATCCGGTTATCATTAATTATTTAATCATTAGTTAGCAAATATTTTTCTTTAATCTTGGCTTATCTCGACAAACTGTATACCTTGTACTAATTATTCGTCTATTTATTTTTTCCTATTATTATGTATCCCTATCTCAGGTGCGTCGTTTTAATTATAGTACCTACGACAATAGTTTACAACAAAGGTATAAGTTACTATGGAAATAGTCTGAAGTTGATAAAACAACGACTAGTCTCTACCTGCAGTAGTTTGTACACAATATTAATATTCTGGTTTCATCCGACTTATTGAATTACGTCTCTATTACATACAATGTCTTTATCTAACACTGTAGGGTTATTTTATAACTAGGTGCCACCTGCGGTTTAATTCTCAACTCATATTGCGCTCACATAAAAATTAGCATCGATAATAATGTGGCTACTTAACACTACAAGAATTTCTCAAATTACCCCAGCTATCCCAGGTTCAGATATAATGGTTCAGGCATAGACGATAATGATAACTGAGATACTTTTTTATGTTCTAAAGTACTTTTCCATCTTGTTCCAGGTGTACGCTTCGCTCGCCTCCAAGTGAAAGCGGGTCTCGCCTGGCTGTTACGTCGTTTTACCTTAGTCGAACAGGACAATTGCATACCAGAATTCGAGAAAAGTCCATTTGGCCTCCGAAGTCCCGATGCTTGTTATGAATTAAAGTTAAGAGATATATAA

>CYP337B1-BF1 of Helicoverpa armigera from Burkina Faso (strain: BF) GTACCATGGAATCACCATTCGTTGAATTGGCATTTAGAGCTTTACGACCATCGTTTATTCAAAATGTAATAGTTGTTATTGCAAATACTTTCCCTAGATTGTACAAACTCTTGCAGTTGAAACTTTTTGGTGAACATGAAGAATTTTTTGTGGGTGCAGTGAAAACTGTTTTAGAAAGTAGGAGGCATGATACTACTAAAAGATATGATTTTATTGAGATTTGCTTGGAACTACAGAAGAATGGAATAATGCAGGACTTCTCAACTGGATATAAATTGGAGCCGACCGACGAACTGATGGCTGCTCAAGCATTCTTCTTTTTTGTGGCTGGTGCAGACACCTCTGCTAACACAATGCATTATTCTTTGTTAGAGCTGTCAAACAATCCAAAAATCTTGGCAAAACTACATGAGGAAATTGACAAAGTTTTCGAAGGCGGAGCTGGGGAACTAACTTATAATGACATTGATAAGTTACAATATTTAGATCAGGTTATAAATGAAGCAATGAGAAAGTATCCTCCAGTAGGTGTCATGCAAAGATTGTGCACTAAAGATACAGTTTTACCTTCTGGTATACCCATAGCAAAGGGTAACACAATAATGATTCCAGTGTTCGGTCTTCACAGAGACGAAAAATACTTTGATGACCCACATGTATTTGATCCGGATAGATTTTTACCAGAAAACGTATCCAAAATCAAGAATTATGCTTATTTACCTTTTGGTGAAGGAAACCGCATTTGTATTGGTGAGTATTAAACATTTACTTATACATATAATATATTTTTGTAACATTTGTAACATTTAAGGTTCTAGAGAAGGCATAAATCTTATTTAGCTTTGATCAGTTATTCTAAAGAAGGTACCTAGTATGCAATGGGCGTGCTAGTCTTTTTATGGCCCAAAACAATTGTTCAATGATCGACCATAATCATCAATACTTATTCCACCCCATTCATTCAATCATTGTCTCATATTAAGAGTCATAAAAATACATAAATGAATAGTTATTATTCCCTTTGTTTAGTTTTACTAATTGCATCATCCGATAAATGAGTGAGTGATCTGATAATGCATTATTTAATTTAAATGATAATGACGGCCTAATTCGGTTATCATTAATTATTTAATCGTTAGTTAGCAAATATTTTTCTTTAATCTTGGCTTATCTTGACAAACTGTATACCTAGTACTAATTGTTTGTCTGTTTATTATTACCAATTATTATTTATCCCTATCTCAGGTGTGTTGTTTTAATAATAGTAGGTACCTACGACAATAGTTTACCACAAAGTTATAAGTACTATGTAAATAGTCTGCAGTTGATAAAACAACTACTAGTCTCTACCTGAGTGCAGTGGTTTGTACACAATACCTATTAATATTCTAGTTTCATCTGACTTATTGAATGACGTCCCTATTACACACAATGTCGTTATCTAACACTGTAGGTTTATTTTATAACTAGGTTCCGCCAGCAGTTTAACTCTCAACCCACACATAAAAGTAGCATCAATAATAATGTGGCTATTTAACACTGAAAAAAAATCTCAAATTACCTCAGCTATCCCAGGTTCAGATATAATGGTTCAGGTATAGATGATGATGATACCTGAGGTATTTTTTATGTTCTAAGGTACGATTCCATCTTGTTCCAGGTGTACGCTTCGCTCGCCTCCAAGTGAAAGCGGGTCTCGCCTGGCTGTTACGTCGTTTTACATTAGCCGAACAGGACAATTGCATACCAGAATTCGAGAAAAGTCCATTTGGCCTCCGAAGTCCCGATGCTCGTTATGAATTAAAGTTAAGAGACATATAA

>CYP337B1AN02v1like

GTACCATGGAATCACCATTCGTTGAATTGGCATTTAGAGCTTTACGACCATCGTTTATTCAAAATGTAATAGTTGTTATTGCAAATACTTTCCCTAGATTGTACAAACTCTTGCAGTTGAAACTTTTTGGTGAACATGAAGAATTTTTTGTGGGTGCAGTGAAAACTGTTTTAGAAAGTAGGAGGCATGATACTACTAAAAGATATGATTTTATTGAGATTTGCTTGGAACTACAGAAGAATGGAATAATGCAGGACTTTTCAACTGGATATAAATTGGAGCCGACCGACGAACTGATGGCTGCTCAAGCATTCTTCTTTTTTGTGGCTGGTGCAGACACCTCTGCTAACACAATGCATTATTCTTTGTTAGAGCTGTCAAACAATCCAAAAATCTTGGCAAAACTACATGAGGAAATTGACAAAGTTTTCGAAGGCGGAGCTGGGGAACTAACTTATAATGACATTGATAAGTTACAATATTTAGATCAGGTTATAAATGAAGCAATAAGAAAGTATCCTCCCATAGGTGTCATGCAAAGATTGTGTACTAAAGATACAGTTTTACCGTCTGGTATACCCATAGCAAAGGGTAACACAATAATGATTCCAGTGTTCGGTCTTCACAGAGATGAAAAATACTTCGATGACCCACATGTATTTGATCCGGATAGATTTTTACCAGAAAACGTATCCAAAATCAAGAATTACGCTTATTTACCTTTTGGTGAAGGAAACCGCATTTGTATTGGTGAGTATTAAACATTTATACATATGTATTGTTAATATTGTAAATATATATGTGTAACTCATACATTTAAGATTGTAGTGAAGGCATAAATCTTATTTAGCTTTGATCAGTTATTCTGAAGTAGGTACCTAGTACGCAATGGGCGTGCTAGTCTTTTTATGGCCCAAAACAATTGTTCAATGAACGGTCATAATCTTCCATACTTATTCCACCCCATTCATTCAATCATTGTCTCATAGTAAGTAGTCATAAAAATACATAAATGAATAGTTATCATTCCCTTTGTTTAGTTTTACTAATTGCATCATCCGATAAATCAGTGAGTGATCTGATAATGCATTATTTAATTTAAATGATAATGACGGCCTAATCCGGTTATCATTAATTATTTAATCATTAGTTAGCAAATATTTTTCTTTAATCTTGGCTTATCTCGACAAACTGTATACCTTGTACTAATTATTCGTCTATTTATTTTTTCCTATTATTATGTATCCCTATCTCAGGTGCGTCGTTTTAATTATAGTACCTACGACAATAGTTTACAACAAAGGTATAAGTTACTATGGAAATAGTCTGAAGTTGATAAAACAACGACTAGTCTCTACCTGCAGTAGTTTGTACACAATATTAATATTCTGGTTTCATCCGACTTATTGAATTACGTCTCTATTACATACAATGTCTTTATCTAACACTGTAGGGTTATTTTATAACTAGGTGCCACCTGCGGTTTAATTCTCAACTCATATTGCGCTCACATAAAAATTAGCATCGATAATAATGTGGCTACTTAACACTACAAGAATTTCTCAAATTACCCCAGCTATCCCAGGTTCAGATATAATGGTTCAGGCATAGACGATAATGATAACTGAGATACTTTTTTATGTTCTAAAGTACTTTTCCATCTTGTTCCAGGTGTACGCTTCGCTCGCCTCCAAGTGAAAGCGGGTCTCGCCTGGCTGTTACGTCGTTTTACCTTAGTCGAACAGGACAATTGCATACCAGAATTCGAGAAAAGTCCATTTGGCCTCCGAAGTCCCGATGCTTGTTATGAATTAAAGTTAAGAGACATATAA

>CYP337B1AN02v4like

GTACCATGGAATCACCATTCGTTGAATTGGCATTTAGAGCTTTACGACCATCGTTTATTCAAAATGTAATAGTTGTTATTGCAAATACTTTCCCTAGATTGTACAAACTCTTGCAGTTGAAACTTTTTGGTGAACATGAAGAATTTTTTGTGGGTGCAGTGAAAACTGTTTTAGAAAGTAGGAGGCATGATACTACTAAAAGATATGATTTTATTGAGATTTGCTTGGAACTACAGAAGAATGGAATAATGCAGGACTTTTCAACTGGATATAAATTGGAGCCGACCGACGAACTGATGGCTGCTCAAGCATTCTTCTTTTTTGTCGCTGGTGCAGACACCTCTGCTAACACAATGCATTATTCTTTGTTAGAGCTGTCAAACAATCCAAAAATCTTGGCAAAACTACATGAGGAAATTGACAAAGTTTTCGAAGGTGGAGCTGGGGAACTAACTTATAATGACATTGATAAGTTACAATATTTAGATCAGGTTATAAATGAAGCAATGAGAAAGTATCCTCCAGTAGGTGTCATGCAAAGATTGTGTACTAAAGATACAGTTTTACCTTCTGGTATACCCATAGCAAAGGGTAACACAATAATGATACCAGTGTTCGGTCTTCACAGAGACGAAAAATACTTTGATGACCCACACGTATTTGATCCGGATAGATTTTCACCAGAAAACGTATCCAAAATCAAGAATTATGCTTATTTACCTTTTGGTGAAGGAAACCGCATTTGTATTGGTGAGTACCTATTAAACATCTATACATATGTATTGTTAATCTTCATATATATAAAAATGAATTGCTGTTCGTTAGTCTTACTAAAACTCGAGTATGGCTGGACCGATTTGGCTTATTTTGGTGTTAAAATGTTTGTAGAGGTCCAGGGAAGGTTTAAACGATACGAAGTTTGCGGGATCAGCTAGTATTGTAAATATATTTGTGTAACTTATACTTATTTAGCTTTGATCAGTTATTCTAAAGAAGGTACCTATTACGCAATGTGCGTCCTAGTCTTTTTATGGATCAAAACAATTGTTCAATGAACGGTCATAATCTTCAATACTTATTCTACCCCACTCATTCAATCATTGTCTCATATTAAGAAGTCATAAAAATACATAAATGAATAGTTATCATTCCCTTTGTTTAGTTTTACTAATTGCATCATCCGATAAATGAGTTAGTGATCTGATAATGCATTATTTAATTTAAATTATAATGACGGCCTAATTCGGTTATCATTAATCATATTAATAATTAGTTAGCAAATATTTTTCCTTAATCTTGGCTTATCTTGACAATCTTTATACCAAGTAAAATTGTTTGTTTGTTTATTTTACCTATTACTATTATACCTATTACTATTTATCCCTATTTATTATCTCAAGTGCGTATTTGTTTAATAGTAGTATTTACGACAATAGTTTACCACAAAGTTATATAAATACTATGTAAATAGTCTGAAGTTGACAAAACAGCGACTAGTCTACCTAAGTGCAGTGGTTTGTACACAATACCTATTAATATTCTAGTTTCATCTGACTTATTGAATTACGTCCCTATTACATACAAGGTCTTTATCTAACACTGTAGTGTTATTTTATAACTAGCTTCCGCCAGCGGTTTAACTCTCAACCTACACATAAAAGTAGCATCAATAATAATATGGCTACTTAATAATATATTAATAAGAATTTCTCAAATCACCCTAGTTATCCCAGGTTAAAATATTAGTATAGATGATAGTGAAAACTGAGATATTTTTTATGTTCTAAAGTACTTTTCTATCTTGTTCCAGGTGTACGCTTCGCTCGCCTCCAAGTGAAAGCGGGTCTCGCCTGGCTGTTGCGTCGTTTTACCTTAGTTGAACAGGACAATTGCGTACCAGAATTCGAGAAAAGTCCTTTCGGTCTACGAAGCCCCGATGCTCGTTATGAATTAAAGTTAAGAGACATATAA

>GRCYP337B1-GR3intron

GTACCATGGAATCACCATTCGTTGAATTGGCATTTAGAGCTTTACGACCATCGTTTATTCAAAATGTAATAGTTGTTATTGCAAATACTTTCCCTAGATTGTACAAACTCTTGCAGTTGAAACTTTTTGGTGAACATGAAGAATTTTTTGTGGGTGCAGTGAAAACTGTTTTAGAAAGTAGGAGGCATGATACTACTAAAAGATATGATTTTATTGAGATTTGCTTGGAACTACAGAAGAATGGAATAATGCAGGACTTCTCAACTGGATATAAATTGGAGCCGACCGACGAACTGATGGCTGCTCAAGCATTCTTCTTTTTTGTGGCTGGTGCAGACACCTCTGCTAACACAATGCATTATTCTTTGTTAGAGCTGTCAAACAATCCAAAAATCTTGGCAAAACTACATGAGGAAATTGACAAAGTTTTCGAAGGTGGAGCTGGGGAACTAACTTATAATGACATTGATAAGTTACAATATTTAGATCAGGTTATAAATGAAGCAATGAGGAAGTATCCTCCAGTAGGTGTCATGCAAAGATTGTGTACTAAAGATACAGTTTTACCTTCTGGTATACCCATAGCAAAAGGTAACACGATAATGATTCCAGTGTTCGGTCTTCACAGAGACGAAAAATACTTCGATGACCCACATGTATTTGATCCGGATAGATTTTTACCAGAAAACATATCCAAAATCAAGAATTATGCTTATTTACCTTTTGGTGAAGGAAACCGCATTTGTATCGGTGAGTATTAGATATTTATACATATGTATTGTTAATATTGTAAATATATTTGAGTAACTTTTACATATTTAAAGTTCTAGAGAAGGCATAAATCTTATTTAGCTTTGATGAGTTATTCTAAAGAAGGTACCTAGTATGCAATGGGCGTGCTAGTCTTTTTATGGTCCAAAACAATTGTTCAATGAACAGCCATAATTTTCAATTCTTATTCCACCCCATTCATTCAATCATTGTCTCATAAAGTAGTCATAAAAATACATAAATGAATTATAATAGTTATCATTCCTTTTGTTTAGTTTTACTAATTGCATCATCCGATAAATGAGTTAGTGATCTGATAATGCATTATTTAATTTAAATGATAATGACGGCCTAATTCGGTTATCATAAATCATTTAATCATTAGTTAGCAAATATTTTTCTTTAATCTTGGCTTATCTTGACAAAATACCTAGTACTAATTGTTTGTCTGTTTATTATTACCTATTATTATTTATCCCCATCTCAGGTGCGTTGTTTTAATAATAGTACCTACGACAAAAGTTTACAACAAAGGTATAAGTTACTATGGAACTATTTCCATAGTAACTTATACGTTGTTTTATCAACTTCAGACTATTCTGAAGTTGATAAAACAACGACTAGTCTCTACCTGCAGTGGTTTGTACACAATATTAATATTCTGGTTTCATTTGACTGATTGAATTACGTCCCTATTACATACAAGGTCTCTATCTAACACTGTAGGATTATTTTATAACTAGCTGCCGCCAGCAGTTTAACTCTCAACCCACATAAAAGTAGCATAGATTATAATGTGGCTGCTTCACACTTTAAGAATTTCTCAAATTACCCCAGCTAAGTATGCCAGGTTCAGATATAATGGTCCAGGTATAGATGATAATGATAAGTCAGATATTTTTTATGTTCTAAAGTACTTTTACATCTTGTTCCAGGTGTACGCTTCGCTCGCCTCCAAGTGAAAGCGGGTCTCGCCTGGCTGTTGCGTCGGTTTACTTTAGTCGAACAGGACAATTGCATACCAGAATTCGAGAAAAGTCCATTTGGCCTCCGAAGTCCCGATGCTCGTTATGAATTAAAGTTAAGAGACATATAA

>GRCYP337B1-GR2intron

GTACCATGGAATCACCATTCGTTGAATTGGCATTTAGAGCTTTACGACCATCGTTTATTCAAAATGTAATAGTTGTTATTGCAAATACTTTCCCTAGATTGTACAAACTCTTGCAGTTGAAACTTTTTGGTGAACATGAAGAATTTTTTGTGGGTGCAGTGAAAACTGTTTTAGAAAGTAGGAGGCATGATACTACTAAAAGATATGATTTTATTGAGATTTGCTTGGAACTACAGAAGAATGGAATAATGCAGGACTTCTCAACTGGATATAAATTGGAGCCGACCGACGAACTGATGGCTGCTCAAGCATTCTTCTTTTTTGTGGCTGGTGCAGACACCTCTGCTAACACAATGCATTATTCTTTGTTAGAGCTGTCAAACAATCCAAAAATCTTGGCAAAACTACATGAGGAAATTGACAAAGTTTTCGAAGGTGGAGCTGGGGAACTAACTTATAATGACATTGATAAGTTACAATATTTAGATCAGGTTATAAATGAAGCAATGAGGAAGTATCCTCCAGTAGGTGTCATGCAAAGATTGTGTACTAAAGATACAGTTTTACCTTCTGGTATACCCATAGCAAAAGGTAACACGATAATGATTCCAGTGTTCGGTCTTCACAGAGACGAAAAATACTTCGATGACCCACATGTATTTGATCCGGATAGATTTTTACCAGAAAACATATCCAAAATCAAGAATTATGCTTATTTACCTTTTGGTGAAGGAAACCGCATTTGTATCGGTGAGTATTAGATATTTATACATATGTATTGTTAATATTGTAAATATATTTGAGTAACTTTTACATATTTAAAGTTCTAGAGAAGGCATAAATCTTATTTAGCTTTGATGAGTTATTCTAAAGAAGGTACCTAGTATGCAATGGGCGTGCTAGTCTTTTTATGGTCCAAAACAATTGTTCAATGAACAGCCATAATTTTCAATTCTTATTCCACCCCATTCATTCAATCATTGTCTCATAAAGTAGTCATAAAAATACATAAATGAATTATAATAGTTATCATTCCTTTTGTTTAGTTTTACTAATTGCATCATCCGATAAATGAGTTAGTGATCTGATAATGCATTATTTAATTTAAATGATAATGACGGCCTAATTCGGTTATCATAAATCATTTAATCATTAGTTAGCAAATATTTTTCTTTAATCTTGGCTTATCTTGACAAAATACCTAGTACTAATTGTTTGTCTGTTTATTATTACCTATTATTATTTATCCCCATCTCAGGTGCGTTGTTTTAATAATAGTACCTACGACAAAAGTTTACAACAAAGGTATAAGTTACTATGGAACTATTTCCATAGTAACTTATACGTTGTTTTATCAACTTCAGACTATTCTGAAGTTGATAAAACAACGACTAGTCTCTACCTGCAGTGGTTTGTACACAATATTAATATTCTGGTTTCATTTGACTGATTGAATTACGTCCCTATTACATACAAGGTCTCTATCTAACACTGTAGGATTATTTTATAACTAGCTGCCGCCAGCAGTTTAACTCTCAACCCACATAAAAGTAGCATAGATTATAATGTGGCTGCTTCACACTTTAAGAATTTCTCAAATTACCCCAGCTAAGTATGCCAGGTTCAGATATAATGGTCCAGGTATAGATGATAATGATAAGTCAGATATTTTTTATGTTCTAAAGTACTTTTACATCTTGTTCCAGGTGTACGCTTCGCTCGCCTCCAAGTGAAAGCGGGTCTCGCCTGGCTGTTGCGTCGGTTTACTTTAGTCGAACAGGACAATTGCATACCAGAATTCGAGAAAAGTCCATTTGGCCTCCGAAGTCCCGATGCTCGTTATGAATTAAAGTTAAGAGACATATAA

>GRCYP337B1-GR1

GTACCATGGAATCACCATTCGTTGAATTGGCATTTAGAGCTTTACGACCATCGTTTATTCAAAATGTAATAGTTGTTATTGCAAATACTTTCCCTAGATTGTACAAACTCTTGCAGTTGAAACTTTTTGGTGAACATGAAGAATTTTTTGTGGGTGCAGTGAAAACTGTTTTAGAAAGTAGGAGGCATGATACTACTAAAAGATATGATTTTATTGAGATTTGCTTGGAACTACAGAAGAATGGAATAATGCAGGACTTTTCAACTGGATATAAATTGGAGCCGACCGACGAACTGATGGCTGCTCAAGCATTCTTCTTTTTTGTGGCTGGTGCAGACACCTCTGCTAACACAATGCATTATTCTTTGTTAGAGCTGTCAAACAATCCAAAAATCTTGGCAAAACTACATGAGGAAATTGACAAAGTTTTCGAAGGTGGAGCTGGGGAACTAACTTATAATGACATTGATAAGTTACAATATTTAGATCAGGTTATAAATGAAGCAATGAGAAAGTATCCTCCCGTAGGTGTCATGCAAAGATTGTGCACTAAAGATACAGTTTTACCTTCTGGTATACCCATAGCAAAAGGTAACACGATAATGATTCCAGTGTTCGGTCTTCACAGAGACGAAAAATACTTCGATGACCCACATGTTTTTGATCCGGATAGATTTTTACCAGAAAACGTATCCAAAATCAAGAATTATGCTTATTTACCTTTTGGTGAAGGAAACCGCATTTGTATTGGTGAGTATTAAACATTTATACATATGTATTGTTAATATTGTAAATATATTTGTGTAACTTATACATATTTAAAGTTCTAGAGAAGGCATAAATCTTATTTAGCTTTGATCAGTTATTCTAAAGAAGGTACCTAGTATGCAATGGGCGTGCTAGTCTTTTTATGGCCCAAAACAATTGTTCAATGATCGACCATAATCATCAATACTTATTCTACCCCATTCATTCAATCATTGTCTCATATTAAGAAGTCATAAAAATAAATAAATGAATACTTATCATTCCCTTTGTTTAGTTTTACTAATTGCAACATCCGATAAATCAGTGAGTGATCTGATAATGCATTATTTAATTTAAATTATAATGACGGCCTAATTCGGTTATCATTAATCATTTTAATAATTATTTAGCAAATATTTTTCCTTAATCTTGGCTTATCTTGACAAACTGTATACCTAGTACTAATTGTTTGTCTGTTTATTATTACCTATTATTATTTATCCCTATCTCAGATGTGTTGTTTTAATAATAGTAGCTAGGTACCTACTACACAATAGTTTACCACAAAGTTATAAGTACTATGTAAATAGTCTGCAGTTGATAAAACAACGACTGGTCTCAACCTGAGTGCACTGGTTTGTACACAATATTCTGGTTTCATCTGACTTATTGAATTACGTCCCTATTACATACAATCATACAAGGTCTTTATATAACACTGTAGGGTTATTTTGTAACCAGCTGCCGAAAGCGGTATAACTCTCAACCCACCCATAAAAAGTAGTATCGATATATCGTATCGATAATAATGTGGCTACTTAACACTATAAGAATTTCTCAAATTACCCCCAGCTATCCCTGGTTCAGATATAATGGTTCAGGTATATATCAGGTGATGATGATAACTGAGATATTTTTTATGTTCTAAAGTACTTTTCCGTCTTGTTCCAGGTGTACGCTTCGCTCGCCTCCAAGTGAAAGCGGGTCTCGCCTGGCTGTTACGTCGTTTTACTTTAGTCGAACAGGACAATTGCGTACCAGAATTCGAGAAAAGTCCATTTGGCCTCCGAAGTCCCGATGCTCGTTATGAATTAAAGTTAAGAGACATATAA

>CYP337B3v5

GTACCATGGAATCACCATTCGTTGAATTGGCATTTAGAGCTTTGCGACCATCGTTTATTCAAAATGTAATAGTTGTTATTGCAAATACTTTCCCTAGATTGTACAAACTCTTGCAGTTGAAACTTTTTGGTGAACATGAAGAATTTTTTGTGGGTGCAGTGAAAACTGTTTTAGAAAGTAGGAGGCATGATACTACTAAAAGATATGATTTTATTGAGATTTGCTTGGAACTACAGAAGAATGGAATAATGCAGGACTTCTCAACTGGTTATAAATTGGAGCCAACCGACGAACTGATGGCTGCTCAAGCATTCTTCTTTTTTGTGGCTGGTGCAGACACCTCTGCTAACACAATGCATTATTCTTTGTTAGAGCTGTCAAACAATCCAAAAATCCTGGCAAAACTACACGAAGAAATTGACAAAGTTTTCGAAGGCGGAGCTGGGGAACTAACTTATAATGACATTGATAAGTTACAATATTTAGATCAGGTTATAAATGAAGCAATGAGAAAGTATCCTCCAGTAGGTGTCATGCAAAGATTGTGCACTAAAGATACAGTTTTACCTTCTGGTATACCCATAGCAAAGGGTAACACAATAATGATTCCAGTGTTCGGTCTTCACAGAGACGAAAAATACTTTGATGACCCACATGTATTTGATCCGGATAGATTTTTACCAGAAAACGTATCCAAAATCAAGAATTATGCCTATTTACCTTTTGGTGAAGGAAACCGCATTTGTATTGGTGAGTACTAAACATTTACTTATACATATAATATATTTTTGTAACATTTGTAACATTTAAGGTTCTAGAGAAGGCATAAATCTTATTTAGCTTTGATCAGTTATTCTAAAGAAGGTACCTAGTATGCAATGGGCGTGCTAGTCTTTTTATGGCCTAAAGCAATTGTTCAATGATCGACCATAATCATCAATACTTATTCCACCCCATTCATTCAATCATTGTCTCATATTAAGAGTCATAAAAATACATAAATGAATAGTTATCATTCCCTTTGTTTAGTTTTACTAATTGCATCATCCGATAAATGAGTGAGTGATCTGATAATGCATTATTTAATTTTTTAATTTAAATGATAATGACGGCCTAATTCGGTTATCATTAATCATTTTAATCATTAGTTAGCAAATATTTTTCTTTAATCTTGGCTTATCTTGACAATCTTTATACCAAGTAAAATTGTTTGTTTGTTTATTTTTACCTATAACTATTTATCCCTATTTATTATCTCAAGTGCGTATTTGTTTAATAGTAGTATTTACGACAATAGTTTACCACAAAGTTATATAAGTACTATGTAAATAGTCTGCAGTTGATAAAACAACTACTAGTCTCTACCTGAGTGTAGTGGGTTGTACACAATATTATTATTCTGGGTTTCATCTGACTTATTGAATTACGTCGCTATAATAAACAAGGTCTAGCACTAGATCTAGCACTGTACCGTTATATTATAACTAGCTGCTGCCAGCAGTTTAACTCTAAACCCATATCACGCACACATATAAATAGTTGCATCGATAATAATGTGGCTATTTAACACTGAAAAAAATTCTCGAATCACCCCAGTTATTCCAGGTTGAAGCAAACAAACTTTTCAGCTTTAAAATATTAGTCATCTACTAATATTTTAATAATGATATTTGAGATATTTTTTATGTTCTAAAGTACTTTTACATCTTGTTCCAGGTGTACGCTTCGCTCGCCTCCAAGTGAAAGCGGGTCTCGCCTGGCTGTTGCGTCGTTTTACTCTAGTCGAACAGGACAATTGCGTACCAGAATTCGAGAAAAGTCCATTCGGTCTACGAAGTCCCGATGCTCGTTATGAATTAAAGTTAAGAGACATATAA

>CYP337B3v6

GTACCATGGAATCACCATTCGTTGAATTGGCATTTAGAGCTTTACGACCATCGTTTATTCAAAATGTAATAGTTGTTATTGCAAATACTTTCCCTAGATTGTACAAACTCTTGCAGTTGAAACTTTTTGGTGAACATGAAGAATTTTTTGTGGGGGCAGTGAAAACTGTTTTAGAAAGTAGGAGGCATGATACTACTAAAAGATATGATTTTATTGAGATTTGCTTGGAACTACAGAAGAATGGAATAATGCAGGACTTTTCAACTGGATATAAATTGGAGCCGACCGACGAACTGATGGCTGCTCAAGCATTCTTCTTTTTTGTGGCTGGTGCAGACACCTCTGCTAACACAATGCATTATTCTTTGTTAGAGCTGTCAAACAATCCAAAAATCTTGGCAAAACTACATGAGGAAATTGACAAAGTTTTCGCAGGTGGAGCTGGGGAACTAACTTATAATGACATTGATAAGTTACAATATTTAGATCAGGTTATAAATGAAGCAATGAGAAAGTATCCTCCAGTAGGTGTCATGCAAAGATTGTGTACTAAAGATACGGTTTTACCTTCTGGTATACCCATAGCAAAGGGTAACACAATAATGATACCAGTGTTCGGTCTTCACAGAGCCGAAAAATACTTTGATGACCCCCATGTATTTGATCCGGATAGATTTTCACCAGAAACCGTATCCAAAATCAAGAATTATGCTTATTTACCTTGTGGTGAAGGAAACCGCATTTGTATTGGTGAGTACCTATTAAACATCTATACATATGTATTGTTAATCTTCATATATATAAAAATGAATTGCTGTTCGTTAGTCTTACTAAAACTCGAGTATGGCTGGACCGATTTGGCTTATTTTGGTGTTAAAATGTTTGTAGAGGTCCAGGGAAGGTTTAAACGATACGAAGTTTGCGGGATCACCTAGTATTGTAAATATATTTGTGTAACTTATACTTATTTAGCTTTGATCAGTTATTCTAAAGAAGGTACCTATTACGCAATGTGCGTCCTAGTCTTTTTATGGATCAAAACAATTGTTCAATGATCGACCATAATGATCACTACTTATTCTACCCCATTCATTCAATCATTGTCTCATAGTAAGTAGTCATAAAAATACATAAATGAATGGTATCATTCCCTTTGTTTAGTTTTTACATATTGCATCATCCGATAAATGAGTGAGTGATCTGATAATGCATTATTTAATTTAAATGATAGTGACGGCCTAATTCGGTTATCATTAATCATTTAATCATTAGTTAGCAAATATTTTTCTTTAATCTTGGCTTATCTTGACAAAATACCTAGTACTAATTGTTTGTTTGTTTATTTTAACCTATAACTATTTATCCCTATTTATTATCTCAAGTGCGTATTTGTTTAATAGTAGTATTTACGACAATAGTTTACCACAAAGTTATATAAATACTATGTAAATAGTCTGAAGTTGACAAAACAGCGACTAGTTTACCTGAGTGCAGTGGTTTGTACACAATACCTATTAATATTCTGGTTTCATCTGACTTATTGAATTACGTCCCTATTACATACAAGGTCTTTATCTAACACTGTAGTGTTATTTTATAACTAGCTTCCGCCAGCGGTTTAACTCTCAACCTACACATAAAAGTAGCATCAATAATAATGTGGCTATTTAACACTGAAAAAAAATCTCAAATTACCCCAGCTATCCCAGGTTCAGATATAATGGTTCAGGTATAGATGATGATGATACCTTAGGTATTTTTTATGTTCTCAAGTACGATTCCATCTTGTTCCAGGTGTACGCTTCGCTCGCCTCCAAGTGAAAGCGGGTCTCGCCTGGCTGTTACGTCGGTTTACCTTAGTTGAACAGGACAATTGCATACCAGAAATCGAGAAAAGTCCATTTGGTCTACGAAGCCCGGATGCTCGTTATGAATTAAAGTTAAGAGACATATAA

>CYP337B3v4

GTACCATGGAATCACCATTCGTTGAATTGGCATTTAGAGCTTTACGACCATCGTTTATTCAA

AATGTAATAGTTGTTATTGCAAATACTTTCCCTAGATTGTACAAACTCTTGCAGTTAAAACTTTTTGGTG

AACATGAAGAATTTTTTGTGGGTGCAGTGAAAACTGTTTTAGAAAGTAGGAGGCATGATACTACTAAAAG

ATATGATTTTATTGAGATTTGCTTGGAACTACAGAAGAATGGAATAATGCAGGACTTCTCAACTGGATAT

AAATTGGAGCCGACTGACGAACTGATGGCTGCTCAAGCATTCTTCTTTTTTGTGGCTGGTGCAGACACCT

CTGCTAACACAATGCATTATTCTTTGTTAGAGCTGTCAAACAATCCAAAAATCTTGGCAAAACTACATGA

GGAAATTGACAAAGTTTTCGAAGGCGGAGCTGGGGAACTAACTTATAATGACATTGATAAGTTACAATAT

TTAGATCAGGTTATAAATGAAGCAATGAGAAAGTATCCTCCAGTAGGTGTCATGCAAAGATTGTGTACTA

AAGATACAGTTTTACCTTCTGGTATACCCATAGCAAAGGGTAACACAATAATGATTCCAGTGTTCGGTCT

TCACAGAGACGAAAAATACTTTGATGACCCACACGTATTTGATCCGGATAGATTTGCACCAGAAAACGTA

TCCAAAATCAAGAATTATGCTTATTTACCTTTTGGTGAAGGAAACCGCATTTGTATTGGTGAGTATTCAA

CATTTACTTATAACTTAATTTAAATATATTTTTGTAACTTTATATTTAAGGTTCTAGAGAAGGCATAAAT

CTTATTTAGCTTTGATCAGTTATTCTAAAGTAGGTACCTAGTACGCAATGGGCGTGCTAGTCTTTTTATG

GCCCAAAACAATTGTTCAATGAACGGTCATAATCTTCCATACTTATTCCACCCCATTCATTCAATCATTG

TCTCATATTAAGTAGTCATAAAAATACATAAATGAATAGTTATCATTCCCTTTGTTTAGTTTTACTAATT

GCATCATCCGATAAATCAGTGAGTGATCTGATAATGCATTATTTAATTTAAATGATAATGACGGCCTAAT

TCGGTTATCATTAGTTATTTAATCGTTAGTTAGCAAATATTTTTCTTTAATCTTGGCTTATCTTGACAAA

CTTTATACCTAGTACTAATTGTTTGTCTGTTTATTTTTACCTATTATTATGTATCCCTATCTCAGGTGCG

TTGTTTTAAAAATAGTATTTACGACAATAGTTTACCACAAAGTTATATAATGCACTATGTAAATAGTCTG

AAGTTGATAAAACAACGACTAGTCTACATGAGTGCAGTGGTTTGTACACAATATCTATTAATATTCTGGT

TTCATCTGACTTATTGAATTACGTCGCTATAACATACAAGGTTTTTATCTAATACTGTACGGTTATATTA

TAACTAGCTTCCGCCAGTGGTTTAACTCTTAACCCACACATAAAAAGTAGCATCGATTATAATGTTGCTG

CTTAACACTTTAAGAATTTCTCAAATTACCCCAGCTATCCCAGGTTCAGATATAATGGTTCAGGTATAGA

TGATGATGATAACTGAGATACTTTTTTTTATGTTTTAAAGTACTTTTCAATCTTGTTCCAGGTGTACGCT

TCGCTCGCCTCCAAGTGAAAGCGGGTCTCGCCTGGTTGTTACGTCGTTTTACTCTAGTCGAACAGGACAA

TTGCATACCAGAATTCGAGAAAAGTCCATTTGGCCTCCGAAGTCCCGATGCTCGTTATGAATTAAAGTTA

AGAGACATATAA

>CYP337B3v3

GTACCATGGAATCACCATTCGTTGAATTGGCATTTAGAGCTTTACGACCATCGTTTATTCAA

AATGTAATAGTTGTTATTGCAAATACTTTCCCTAGATTGTACAAACTCTTGCAGTTGAAACTTTTTGGTG

AACATGAAGAATTTTTTGTGGGTGCAGTGAAAACTGTTTTAGAAAGTAGGAGGCATGATACTACTAAAAG

ATATGATTTTATTGAGATTTGCTTGGAACTACAGAAGAATGGAATAATGCAGGACTTCTCAACTGGATAT

AAATTGGAGCCGACCGACGAACTGATGGCTGCTCAAGCATTCTTCTTTTTTGTGGCTGGTGCAGACACCT

CTGCTAACACAATGCATTATTCTTTGTTAGAGCTGTCAAACAATCCAAAAATCTTGGCAAAACTACATGA

GGAAATTGACAAAGTTTTCGAAGGCGGAGCTGGGGAACTAACTTATAATGACATTGATAAGTTACAATAT

TTAGATCAGGTTATAAATGAAGCAATGAGAAAGTATCCTCCAGTAGGTGTCATGCAAAGATTGTGTACTA

AAGATACAGTTTTACCTTCTGGTATACCCATAGCAAAGGGTAACACAATAATGATTCCAGTGTTCGGTCT

TCACAGAGACGAAAAATACTTTGATGACCCACACGTATTTGATCCGGATAGATTTGCACCAGAAAACGTA

TCCAAAATCAAGAATTATGCTTATTTACCTTTTGGTGAAGGAAACCGCATTTGTATTGGTGAGTATTCAA

CATTTACTTATAACTTAATTTAAATATATTTTTGTAACTTTATATTTAAGGTTCTAGAGAAGGCATAAAT

CTTATTTAGCTTTGATCAGTTATTCTAAAGTAGGTACCTAGTACGCAATGGGCGTGCTAGTCTTTTTATG

GCCCAAAACAATTGTTCAATGAACGGTCATAATCTTCCATACTTATTCCACCCCATTCATTCAATCATTG

TCTCATATTAAGTAGTCATAAAAATACATAAATGAATAGTTATCATTCCCTTTGTTTAGTTTTACTAATT

GCATCATCCGATAAATCAGTGAGTGATCTGATAATGCATTATTTAATTTAAATGATAATGACGGCCTAAT

TCGGTTATCATTAGTTATTTAATCGTTAGTTAGCAAATATTTTTCTTTAATCTTGGCTTATCTTGACAAA

CTTTATACCTAGTACTAATTGTTTGTCTGTTTATTTTTACCTATTATTATGTATCCCTATCTCAGGTGCG

TTGTTTTAAAAATAGTATTTACGACAATAGTTTACCACAAAGTTATATAATGCACTATGTAAATAGTCTG

AAGTTGATAAAACAACGACTAGTCTACATGAGTGCAGTGGTTTGTACACAATATCTATTAATATTCTGGT

TTCATCTGACTTATTGAATTACGTCGCTATAACATACAAGGTTTTTATCTAATACTGCACGGTTATATTA

TAACTAGCTTCCGCCAGTGGTTTAACTCTCAACCCACACATAAAAAGTAGCATCGATTATAATGTTGCTG

CTTAACACTTTAAGAATTTCTCAAATTACCCCAGCTATCCCAGGTTCAGATATAATGGTTCAGGTATAGA

TGATGATGATAACTGAGATACTTTTTTTTATGTTTTAAAGTACTTTTCAATCTTGTTCCAGGTGTACGCT

TCGCTCGCCTCCAAGTGAAAGCGGGTCTCGCCTGGTTGTTACGTCGTTTTACTCTAGTCGAACAGGACAA

TTGCATACCAGAATTCGAGAAAAGTCCATTTGGCCTCCGAAGTCCCGATGCTCGTTATGAATTAAAGTTA

AGAGACATATAA
